# Antibody cross-reactivity accounts for widespread appearance of m^1^A in 5’UTRs

**DOI:** 10.1038/s41467-019-13146-w

**Published:** 2019-11-12

**Authors:** Anya V. Grozhik, Anthony O. Olarerin-George, Miriam Sindelar, Xing Li, Steven S. Gross, Samie R. Jaffrey

**Affiliations:** Department of Pharmacology, Weill Cornell Medicine, New York, NY 10065 USA

**Keywords:** Data processing, RNA modification

## Abstract

*N*^1^-methyladenosine (m^1^A) was proposed to be a highly prevalent modification in mRNA 5’UTRs based on mapping studies using an m^1^A-binding antibody. We developed a bioinformatic approach to discover m^1^A and other modifications in mRNA throughout the transcriptome by analyzing preexisting ultra-deep RNA-Seq data for modification-induced misincorporations. Using this approach, we detected appreciable levels of m^1^A only in one mRNA: the mitochondrial *MT-ND5* transcript. As an alternative approach, we also developed an antibody-based m^1^A-mapping approach to detect m^1^A at single-nucleotide resolution, and confirmed that the commonly used m^1^A antibody maps sites to the transcription-start site in mRNA 5’UTRs. However, further analysis revealed that these were false-positives caused by binding of the antibody to the m^7^G-cap. A different m^1^A antibody that lacks cap-binding cross-reactivity does not show enriched binding in 5’UTRs. These results demonstrate that high-stoichiometry m^1^A sites are exceedingly rare in mRNAs and that previous mappings of m^1^A to 5’UTRs were the result of antibody cross-reactivity to the 5’ cap.

## Abstract (web summary)

*N*^1^-methyladenosine (m^1^A) was recently reported as a new mRNA modification but its prevalence has been controversial. Here the authors showed that m^1^A, if present in mRNA, is at very low stoichiometry, with the notable exception of *MT-ND5*. Further, they show that the previously reported enrichment of m^1^A near the start of transcripts are false-positive identifications due to cross-reactivity of the commonly used m1A antibody with mRNA caps.

## Introduction

The initial concept of the epitranscriptome was born with the transcriptome-wide mapping of thousands of internally located modified nucleotide *N*^6^-methyladenosine (m^6^A) residues in the transcriptome^1,2^. Two studies later identified *N*^1^-methyladenosine (m^1^A) as another abundant epitranscriptomic modification^3,4^. Both studies mapped m^1^A in thousands of mRNAs by sequencing mRNA fragments immunoprecipitated with a monoclonal antibody (clone AMA-2) commercially distributed by MBL Bioscience, which was originally raised against KLH-conjugated 1-methyladenosine^5^. This antibody was previously shown to recognize m^1^A-containing RNAs^6^. One study estimated the average stoichiometry of mapped m^1^A sites at 20%^3^. Notably, most m^1^A sites were located near start codons and proposed to provide a novel form of translational regulation^3^.

Subsequent work reported different distributions for m^1^A, one arguing that m^1^A was exceptionally rare in mRNA^7^. In that study, the antibody-bound RNA was reverse transcribed with an enzyme that efficiently introduces misincorporations at m^1^A. Using this approach, m^1^A was rarely observed in the RNA immunoprecipitated with m^1^A antibodies^7^. Although mRNA fragments from 5′UTRs and start codon-proximal regions were immunoprecipitated, these fragments did not generate misincorporations. Thus it was concluded that mRNA fragments from the 5′UTR may be nonspecifically enriched during immunoprecipitation^7^. Ultimately it was concluded that only two mRNAs contained high-confidence m^1^A sites: *C9orf100* and *MT-ND5*, a cytosolic and a mitochondrial mRNA, respectively^7^. Twelve other sites were detected at very low stoichiometry.

The second study mapped m^1^A to 740 sites, 473 of which were in mRNA and lncRNA^8^. In mRNAs, the majority of sites were found in the 5′UTR; 22 of which the authors localized to the first nucleotide of the transcript. Based on this location, it was proposed that m^1^A forms a novel cap structure in which m^1^A immediately follows the 7-methylguanosine (m^7^G) cap of mRNA (m^7^G-ppp-m^1^A). A re-analysis of these data showed that many of the sites that were mapped internally within the 5′UTR were actually transcription-start sites^9^.

It remained unclear why those studies produced divergent m^1^A maps, and if m^1^A exists at transcription-start sites or start codons, or neither, and why these particular sites are so prominent in m^1^A-mapping studies. Additionally, whether m^1^A sites are present with high stoichiometry as initially reported^3^, or low stoichiometry and rare^7^ also remained to be resolved.

Here, to address the question of the prevalence and location of m^1^A in the transcriptome, we used both a high-resolution m^1^A-mapping method as well as a bioinformatic approach, termed “misincorporation mapping”. Misincorporation mapping takes advantage of the ability of m^1^A and numerous other modified nucleotides to induce misincorporations during the reverse transcription step common to most RNA-Seq protocols. By probing several ultra-deep RNA-Seq datasets for such misincorporations, we discovered that very few mRNAs contain misincorporations. Only the *MT-ND5* mitochondrial transcript and the *MALAT1* noncoding RNA generated statistically significant misincorporations, demonstrating the rarity of high stoichiometry m^1^A sites. To understand why misincorporation mapping identified only a few m^1^A sites while m^1^A antibody-based mapping detects many, we mapped m^1^A at high resolution using the same m^1^A-directed antibody used in all previous studies. This mapping recapitulated the selective binding of the AMA-2 m^1^A antibody to transcription-start nucleotides in mRNA. However, we also found that this m^1^A antibody recognizes the m^7^G cap structure, and that m^1^A-independent binding explains why previous maps showed m^1^A in mRNA 5′UTR regions. To further confirm this observation, we demonstrate that a different m^1^A antibody, which we show does not bind the m^7^G cap, produces an m^1^A map that no longer enriches for the 5′ end of mRNAs. Overall, our data demonstrate that (1) m^1^A and other hard stop nucleotides are rare in mRNA; (2) that—with the exception of *MT-ND5*—m^1^A sites have very low stoichiometry; and (3) that cross-reactivity of the AMA-2 m^1^A antibody with 5′ caps leads to false-positive localization of m^1^A to transcription-start nucleotides and start codons.

## Results

### Misincorporation mapping using ultra-deep RNA-seq

Given the inconsistency in the different antibody-dependent m^1^A-mapping methods (Supplementary Fig. 1a–c), we sought to use an antibody-independent approach to detect m^1^A at single-nucleotide resolution in mRNA. For this, we took advantage of existing ultra-deep RNA-seq datasets and the fact that m^1^A is a “hard-stop” modification, meaning it typically arrests cDNA synthesized by standard reverse transcriptases^10,11^ (Supplementary Fig. 1d). However, SuperScript III will read through m^1^A and other hard-stop nucleotides at low frequency, resulting in misincorporations that are variable and sequence dependent^10,11^. Most m^1^A-induced misincorporations are A→T transitions that can be detected by sequencing the cDNA^10,11^. This approach can detect other hard-stop modifications, such as 3-methylcytidine (m^3^C), 3-methyluridine, *N*^2^,*N*^2^-dimethylguanosine, and *N*^6^,*N*^6^-dimethyladenosine since these also produce misincorporations^10^. Therefore, misincorporations can directly localize m^1^A and other hard-stop nucleotides in sequencing data^10,11^.

Misincorporations are difficult to distinguish from sequencing errors using standard next-generation sequencing for two major reasons. First, substantial read depth is required to detect m^1^A since m^1^A typically induces a misincorporation in only approximately 20–30% of the cDNAs generated by SuperScript III (refs. ^10,11^). The misincorporations would therefore be particularly difficult to detect for low stoichiometry m^1^A residues. Second, misincorporations cannot be readily distinguished from stochastic errors originating during PCR amplification or during sequencing. Thus, m^1^A cannot be definitively identified in standard RNA-seq experiments.

**Fig. 1:**
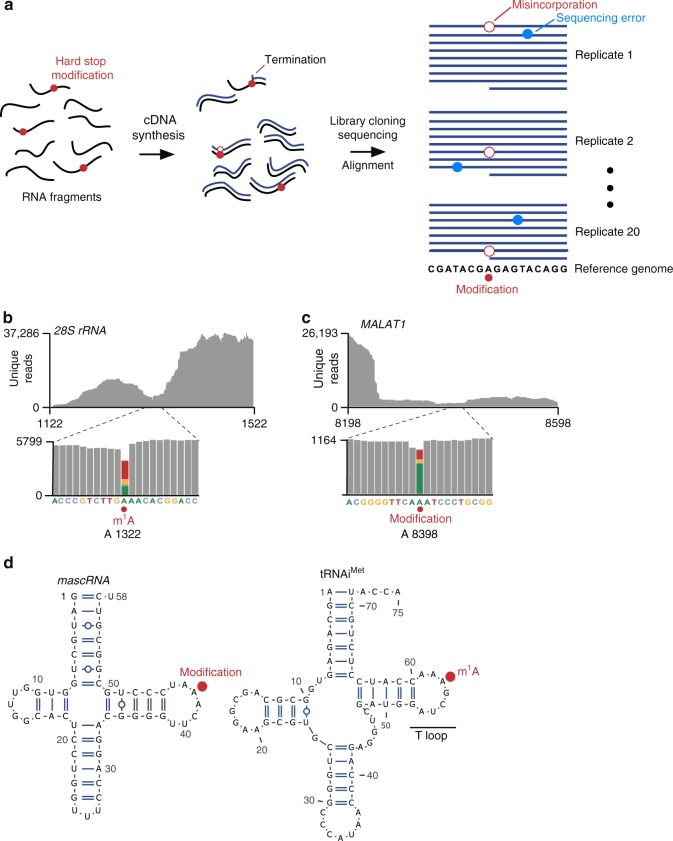
Misincorporation mapping identifies known and novel modifications. **a** Schematic of misincorporation mapping. An ultra-deep RNA-seq dataset was derived from 20 independent biological replicates. Conceivably, some of the RNA fragments (black lines) contain “hard-stop modifications” (red circles), and RNA fragments were reverse transcribed (cDNA, blue lines). For RNA fragments containing these modifications, the reverse transcriptase could terminate at the modification, produce a nucleotide misincorporation, or read through the modification. When the cDNA library is PCR-amplified and sequenced, nucleotide differences are detected between the aligned reads and genomic sequence matching the RNA-seq dataset. Misincorporations (open red circles) identified using this approach are indicative of putative modification sites. Examination of these misincorporations across multiple replicates is critical to distinguish modification sites and sequencing error, which occurs more dispersedly (blue circles). **b** m^1^A in the *28S* rRNA is detected by misincorporation mapping. To determine if known hard-stop nucleotide modifications are detected using misincorporation mapping in ultra-deep RNA-seq, we evaluated mapped sequence reads (gray) around a known m^1^A site in the *28S* rRNA (upper panel). Approximately 70% of the nucleotides that mapped to the m^1^A position contained misincorporations (lower panel; colored bar, position of modified nucleotide and corresponding misincorporations). Additionally, most misincorporations were A→T transitions, typical of m^1^A. **c** Misincorporation mapping detects a modified adenosine in *MALAT1*. To identify novel modified sites, we analyzed misincorporations in lncRNA and mRNA. A high-confidence site was identified in the lncRNA *MALAT1*. More than 25% of reads (gray) mapping to an adenosine in this RNA contained misincorporations (colored bar, position of modified nucleotide and corresponding misincorporations). **d** The modification in *MALAT1* described in **c** occurs in tRNA-like structure at a position that corresponds to the m^1^A position in tRNAs. This region of *MALAT1* is known to be processed to yield a tRNA-like small RNA called *mascRNA*^16^ (left), which is similar to human tRNAs (right, tRNAi^Met^ is shown as an example). The location of the modified adenosine in *mascRNA* is analogous to the position of m^1^A in the T-loop structure of tRNA. This result suggests that the modified adenosine in *mascRNA* is likely to contain m^1^A

To overcome these problems, we developed a bioinformatic approach similar to high-throughput annotation of modified ribonucleotides (HAMR)^10^ to distinguish modified nucleotides from sequencing errors (Fig. 1a). We used an ultra-deep RNA-seq dataset from blood mononucleocytes comprising approximately three billion reads derived from 20 independent sequencing experiments (“replicates”)^12^. These replicates were derived from a single human donor whose genome was sequenced, allowing any differences between the cDNA and genome to be readily detected. As with most RNA-seq datasets, the exact reverse transcriptase termination site is not detectable^12^ (see Supplementary Fig. 1a). Instead, we localized hard-stop modifications by identifying all nucleotide positions in the transcriptome that showed misincorporations across multiple replicates (see Methods). Notably, for m^1^A, we searched for A→T transitions alongside other less common transition types induced by m^1^A^10,11^ (see Methods). Importantly, this method only reveals misincorporations, not the identity of the modification; the identity would have to be determined by biochemical methods.

We first confirmed that we could detect known m^1^A sites. After aligning reads to rRNA, we readily detected the known *28S* rRNA m^1^A at position 1322. (Fig. 1b, Supplementary Fig. 2a). As expected, the misincorporations were predominantly A→T transitions, which are characteristic of m^1^A^10,11^. These site-specific misincorporations were detected in all 20 replicates, confirming that the A→T transitions were not stochastic sequencing errors.

Misincorporation mapping can detect other hard-stop modifications in rRNA, including 1-methyl-3-(3-amino-3-carboxypropyl)pseudouridine and m^3^U (Supplementary Fig. 2b). However, modifications that do not significantly affect reverse transcription, such as m^6^A, pseudouridine, *N*^4^-acetylcytidine, 2′-*O*-methylated nucleotides, and m^7^G, did not induce misincorporations (Supplementary Fig. 2c).

We considered the possibility that m^1^A detection could be impaired because m^1^A can convert to m^6^A through the Dimroth rearrangement, a heat and base-catalyzed reaction^13^ (Supplementary Fig. 2d). To estimate m^1^A loss during the preparation of the ultra-deep sequencing libraries, we examined the m^1^A at position 1322 in the *28S* rRNA, which is methylated at near complete stoichiometry^14^. Since reverse transcription of m^1^A results in read-through approximately 20–30% of the time^10,11^, the fraction of read-through events can suggest the overall m^1^A stoichiometry. Notably, we found that m^1^A at this position was associated with a ~15% read-through rate in this dataset (see Supplementary Fig. 2a). This suggests that the library preparation protocol did not cause substantial degradation of m^1^A, and m^1^A residues should be detectable throughout the transcriptome using this dataset.

### m^1^A is not readily detected in mRNA

In order to detect m^1^A, the modified residue must be reverse transcribed a sufficient number of times during library preparation to generate misincorporations. Since m^1^A sites were reported to have on average a 20% stoichiometry^3^, we set a threshold of 500 unique reads on any given nucleotide to detect m^1^A sites. At this stoichiometry, 100 reverse transcription events would encounter m^1^A. Of these 100 reverse transcription events, approximately 20% would read through, and most of these would be associated with a misincorporation^10,11^. At this read depth, misincorporations should therefore be readily detected in multiple replicates. Thus, to detect m^1^A in mRNA, we restricted our search to approximately eight million adenosine residues in the transcriptome that showed a read depth of >500 reads (Supplementary Fig. 3a).

Analysis of the three billion reads showed 14 high-confidence nucleotide positions across the transcriptome with misincorporations in more than one replicate (see Methods). Of these, 12 occurred at adenosine residues (Supplementary Data 1 and 2). Most of these modified adenosines were found in mitochondrial tRNAs and occurred at known m^1^A positions in mitochondrial tRNAs^15^ (Supplementary Data 2). We also detected a modified adenosine in *mascRNA* (*MALAT1*-associated small cytoplasmic RNA), a short tRNA-like ncRNA that is derived from endonucleolytic processing of *MALAT1* (ref. ^16^) (Fig. 1c). Notably, this modified adenosine corresponds to position 58 within the T-loop of tRNAs (Fig. 1d), a conserved m^1^A site in tRNAs^17^. This m^1^A site in *MALAT1* may be similarly formed by T-loop-specific m^1^A-synthesizing enzymes^17^.

Besides these noncoding RNAs, the previously reported^7^ m^1^A-containing *MT-ND5* mitochondrial mRNA also contained a modified adenosine (Supplementary Data 2). This adenosine exhibited a misincorporation rate of 13.5%. Thus, misincorporation mapping resulted in the same major conclusion as previously reported^7^ that *MT-ND5* is the major m1A-modified mRNA in the cell.

We next examined misincorporations at other mitochondrial mRNAs with previously annotated m^1^A sites. Safra et al.^7^ and Li et al.^8^ identified 11 and 5 putative mitochondrial m^1^A-containing protein-coding genes, respectively. Four mitochondrial mRNAs were common to both studies (*MT-ND5*, *MT-CO1*, *MT-CO2*, and *MT-CO3*). The misincorporation rates in poly(A) RNA-Seq for these mRNAs were very low (less than 0.7% and 2.1% in the Safra and Li studies^7,8^, respectively). However, misincorporations could be detected when m^1^A-containing mRNAs were enriched using the m^1^A antibody, suggesting that m^1^A-containing transcripts are indeed present in cells, but are so rare that they require enrichment to be detected. We therefore leveraged the exceptional mitochondrial read depth in our ultra-deep RNA-seq samples (average ~1 million reads/nucleotide). Here we found that with the exception of *MT-ND5* which had a misincorporation rate of 13.5%, the misincorporation rates for all other putative m^1^A sites was less than 0.4%, which is close to the background rate (Supplementary Fig. 3b, Supplementary Data 3). This demonstrates that the baseline stoichiometry of m^1^A is very low in all mitochondrial mRNAs except for *MT-ND5*, and m^1^A detection requires a pre-enrichment step due to its exceptionally low stoichiometry.

We also detected three additional sites of modifications in cytosolic mRNAs, with only one site being a modified adenosine (Supplementary Data 2). Therefore, it is unlikely that there are many high-stoichiometry hard-stop nucleotides in mRNA.

Notably, when we examined cytosolic mRNAs reported^3^ to have the highest stoichiometry of m^1^A (i.e. >50%) such as *CCDC71*, *DLST*, and *STK16*, each lacked A→T transitions based on misincorporation mapping (Supplementary Fig. 3c, Supplementary Data 1).

m^3^C is also unlikely to be present at high stoichiometry in mRNA since we could not detect any high-confidence misincorporations at cytidine residues (Supplementary Data 2). The m^3^C previously detected in mRNA by mass spectrometry^18^ may therefore have originated from contaminating tRNA.

Although misincorporation mapping demonstrated that few mRNAs have m^1^A or other hard-stop modifications, an important caveat is that the 5′ end of mRNAs cannot be assessed. This is because RNA-seq typically provides less coverage at 5′ ends of RNAs (see Supplementary Fig. 1a)^19^. Overall, the paucity of m^1^A sites in mRNA using misincorporation mapping demonstrates that m^1^A is not a prevalent high-stoichiometry modification in mRNA.

### m^1^A-miCLIP detects known m^1^A sites at nucleotide resolution

**Fig. 2:**
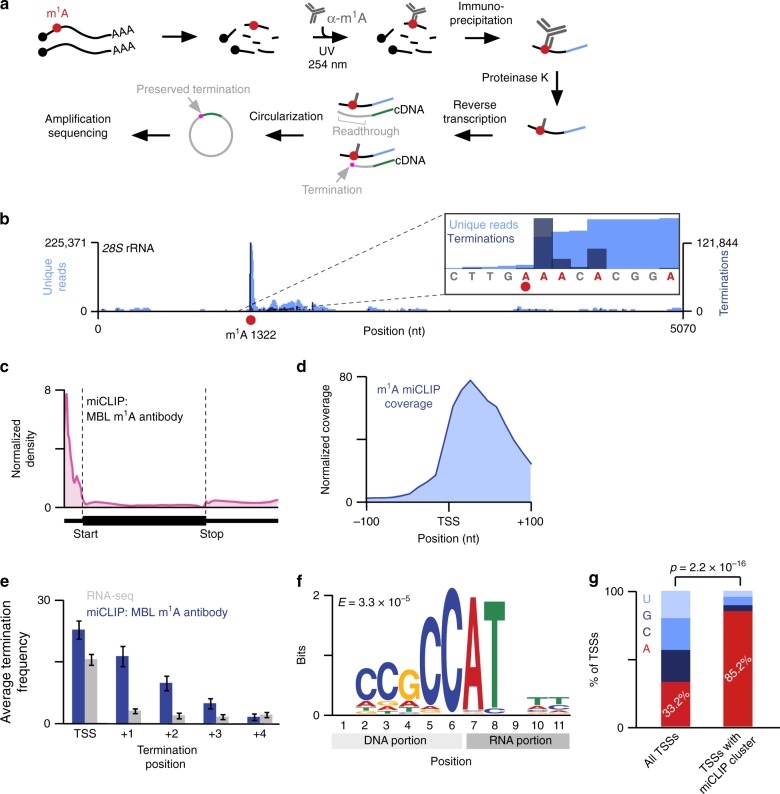
Mapping m^1^A in the transcriptome using m^1^A-miCLIP. **a** m^1^A-miCLIP workflow. Poly(A) RNA (black lines; black circles, RNA caps) was fragmented, incubated with m^1^A antibody, and crosslinked to the antibody with UV. RNA-antibody complexes were immunoprecipitated, ligated to a 3′ linker (blue line), and purified. RNA was released from crosslinked antibody using Proteinase K, generating RNA fragments with peptide adducts. After reverse transcription, first-strand cDNA (gray lines) was circularized to preserve m^1^A-induced read terminations (magenta dot) at the cDNA 3′ end. **b** m^1^A-miCLIP detects the known m^1^A site in the *28S* rRNA. m^1^A-miCLIP unique reads (light blue) were aligned to rRNA showing high enrichment at the m^1^A site (red circle) at position 1322 of the *28S* rRNA. The reads terminated predominantly at the +1 position relative to m^1^A (inset, dark blue). **c** Relative distribution of m^1^A-miCLIP clusters. The metagene distribution of m^1^A-miCLIP clusters that contained 20 or more stacked reads (*n* = 3877) was normalized to RNA-seq coverage and plotted. Clusters were predominantly enriched in the 5′UTR and next to the transcription-start site (start, start codon; stop, stop codon). The annotation of transcription-start sites is not exact, which results in slight coverage upstream of the transcription-start site. **d** m^1^A-miCLIP coverage is enriched at and near transcription-start sites. m^1^A-miCLIP unique reads were mapped relative to annotated transcription-start sites using Deeptools (see Methods). **e** While RNA-seq reads terminate predominantly at position 0 (i.e. due to the end of the transcript), m^1^A-miCLIP reads terminate predominantly at positions 0 and +1, and to a lesser degree, at downstream nucleotides, suggesting that the m^1^A antibody binds at or near the transcription-start site and blocks reverse transcription. Error bars represent SEM. Source data are provided as a Source Data file. **f** m^1^A-miCLIP clusters found in 5′UTRs showed significant enrichment of the pyrimidine-rich Initiator sequence motif that facilitates transcription initiation of mRNAs at adenosine (*E* = 3.3 × 10^−5^). **g** m^1^A-miCLIP clusters were overlapped with a collective set of transcription-start sites (TSS) that included RefSeq annotations and m^6^A_m_ mRNA extended caps^44^. While 33.2% of these collective transcription-start sites were adenosines, the frequency of adenosine transcription-start sites overlapping m^1^A-miCLIP clusters was significantly higher (85.2%, *n* = 1,026 TSSs, *p* < 2.2 × 10^-16^, Fisher’s exact test)

To understand why m^1^A antibody-based mapping approaches produce a prominent 5′UTR signal, we developed an approach to detect m^1^A at single-nucleotide resolution: m^1^A-miCLIP (m^1^A-modification individual nucleotide resolution crosslinking and immunoprecipitation) (Fig. 2a). We initially used the AMA-2 m^1^A antibody previously used to map m^1^A sites in mRNAs^3,4^. In m^1^A-miCLIP, the m^1^A antibody is crosslinked to sheared RNA (Fig. 2a). UV crosslinking with stringent washing reduces nonspecific RNA binding and increases peak resolution in mapping studies^20^. RNA fragments crosslinked to the antibody are then purified and cloned as a cDNA library. Terminations introduced during reverse transcription are then analyzed to localize precise sites where the m^1^A antibody binds throughout the transcriptome. Prior to performing transcriptome-wide m^1^A mapping, we confirmed the AMA-2 m^1^A antibody binds m^1^A, and not other nucleotides (Supplementary Fig. 4a).

m^1^A-miCLIP differs from earlier methods^3,4^ by preserving the cDNA 3′ ends. Any m^1^A antibody-binding site at the transcription-start site would produce a peak that is displaced in the 3′ direction (see Supplementary Fig. 1a). This can make a m^1^A antibody-binding site at the transcription-start site appear to be located at the start codon. To avoid this problem, we chose to generate our libraries in a way that preserves the cDNA ends. Therefore, m^1^A-miCLIP reveals exact m^1^A antibody-binding sites within transcripts.

We performed m^1^A-miCLIP with the AMA-2 antibody using poly(A) RNA from HEK293T cells and examined termination signatures at known m^1^A sites in rRNA and tRNA, which typically co-purify to some extent with poly(A) RNA^21–23^. The majority of reads truncated at the +1 position relative to the m^1^A at position 1322 of the *28S* rRNA (Fig. 2b, Supplementary Fig. 4b) as well as known m^1^A sites in tRNA (Supplementary Fig. 4c). As expected, some read-through was also observed, reflecting the low read-through rate of SuperScript III when it encounters m^1^A. These data demonstrate specific detection of m^1^A by m^1^A-miCLIP using the AMA-2 m^1^A antibody. Notably, m^1^A-miCLIP showed markedly improved peak resolution compared to the initial peak-based m^1^A mapping studies^3,4^ (Supplementary Fig. 4b).

We next asked if m^1^A detection in m^1^A-miCLIP is adversely affected by m^1^A conversion to m^6^A via the Dimroth reaction. m^1^A-miCLIP does not use high temperatures and basic pH^13^ which are needed for this conversion (see Supplementary Fig. 2d). No m^6^A-miCLIP reads^24^ could be detected at the *28S* rRNA m^1^A site (Supplementary Fig. 4d), demonstrating that m^1^A does not appreciably converts to m^6^A during the m^1^A-miCLIP protocol. Taken together, these data demonstrate that m^1^A-miCLIP maps m^1^A with high specificity and resolution.

### The AMA-2 m^1^A antibody binds near the first mRNA nucleotide

We next used m^1^A-miCLIP to map m^1^A in mRNA (Supplementary Fig. 5a). To do this, we aligned m^1^A-miCLIP unique reads to the genome, generated m^1^A-miCLIP clusters (see Methods, Supplementary Data 4), and analyzed their distribution. A metagene analysis of m^1^A-miCLIP clusters obtained from HEK293T cells showed a marked enrichment in the 5′UTR (Fig. 2c). More precisely, the clusters were located at mRNA transcription-start sites (Fig. 2d). A similar enrichment was seen using mouse mRNA (Supplementary Data 5, Supplementary Fig. 5b).

Crosslinking of the m^1^A antibody is expected to cause reverse transcription terminations within several nucleotides of the site of the antibody-RNA adduct^20^. As expected, we found that in miCLIP, terminations were enriched not only at the transcription-start site, but also prominently at the +1 position relative to the transcription-start site (Fig. 2e), with additional terminations sometimes seen between position +2 and +3 (Fig. 2e). Thus, the AMA-2 m^1^A antibody binds at or near mRNA transcription-start nucleotides.

We considered the possibility that terminations near the transcription-start nucleotide could simply reflect general behavior of the reverse transcriptase as it approaches the mRNA 5′ end. To test this, we examined the input RNA fragments in the RNA-seq dataset prepared using the same library cloning strategy as m^1^A-miCLIP. In general, RNA-seq reads terminated almost exclusively at the transcription-start nucleotide (Fig. 2e, Supplementary Fig. 5c). Therefore, read terminations seen in m^1^A-miCLIP near the transcription-start nucleotide are likely induced by selective binding and crosslinking of the AMA-2 m^1^A antibody rather than an artifact of reverse transcription near mRNA 5′ ends.

m^1^A-miCLIP motif analysis revealed a consensus sequence in the upstream genomic region that was pyrimidine-rich (Fig. 2f) and highly similar to Initiator, a transcription-initiating sequence which produces transcripts that initiate with adenosine^25,26^. Indeed, 85% of transcripts containing an m^1^A-miCLIP cluster at their transcription-start nucleotide initiated with adenosine (Fig. 2g, Supplementary Data 6). Thus, we reasoned that adenosine at the transcription-start nucleotide was important for binding of the AMA-2 antibody at or near the transcription-start site.

### m^1^A and m^1^A_m_ are not detected in extended cap structures

Because the AMA-2 m^1^A antibody binds at transcription-start sites, it has been proposed that mRNAs contain a novel mRNA cap structure comprising m^7^G followed by *N*^1^-methylated adenine at the transcription-start nucleotide^8^. The methylated nucleotide would be m^1^A or *N*^1^,2′-*O*-dimethyladenosine (m^1^A_m_) since the first encoded nucleotide of mRNAs is typically subjected to 2′-*O*-methylation^27,28^. To biochemically validate this, we used mass spectrometry to detect m^1^A or m^1^A_m_ in “cap dinucleotides,” i.e., m^7^G-ppp-m^1^A_m_. We treated cellular RNA with P1 nuclease, which digests internal nucleotides to mononucleotides, but leaves the cap dinucleotide intact (see Methods).

**Fig. 3:**
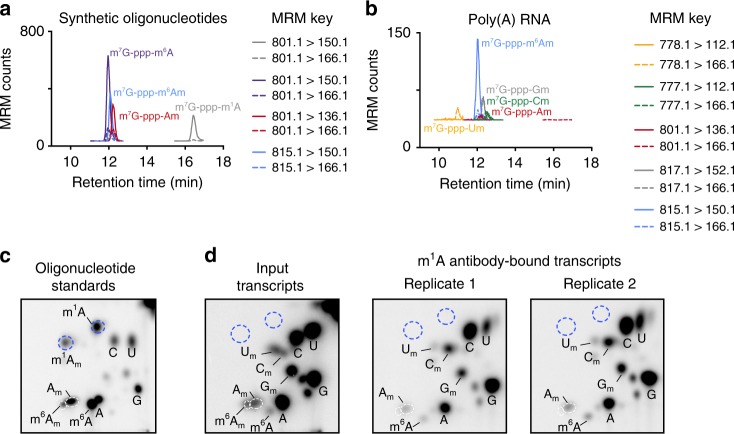
The AMA-2 m^1^A antibody does not enrich for a novel m^1^A-containing cap structure. **a** Detection of synthetic 5′ cap dinucleotides using mass spectrometry. Dynamic multiple reaction monitoring (dMRM) chromatograms were recorded in positive ion monitoring. Injected was a mixture of oligonucleotides containing m^7^G-ppp-m^6^A_m_, m^7^G-ppp-m^1^A, m^7^G-ppp-m^6^A, or m^7^G-ppp-A_m_ cap structures. Two MRM transitions were recorded for each standard (indicated in key in figure). Note the permanent positive charge on the adenine base of m^7^G-ppp-m^1^A resulted in an increased retention time on the ANP resin as expected. Thus, the m^7^G-ppp-m^1^A_m_ dinucleotide would be expected to elute at a retention time later than m^7^G-ppp-m^6^A due to the permanently positive charged adenine, and earlier than m^7^G-ppp-m^1^A due to the additional methyl group on the ribose. **b** Detection of 5′ cap dinucleotides from cellular mRNA using mass spectrometry. Dynamic multiple reaction monitoring (dMRM) chromatograms were recorded in positive ion monitoring. Injected were mRNA samples from HEK293T cells. Two MRM transitions were recorded for each cap structure of interest (indicated in key in figure). We searched for peaks containing MRM transitions and retention times reflecting m^7^G-ppp-m^1^A or m^7^G-ppp-m^1^A_m_ (see note in **a**), but no such signal was detected. **c** 2D-TLC analysis of capped oligonucleotide standards containing various modified adenines as initiating nucleotides. The migration properties of these various initiating nucleotides are indicated (here, the initiating nucleotides include m^1^A, m^1^A_m_, A_m_, m^6^A, and m^6^A_m_). Spots reflecting A, C, G, and U are also marked. Unmarked spots most likely reflect RNA dinucleotides that are not completely digested during RNA preparation^45^. **d** 2D-TLC analysis of mRNA extended caps in antibody-unbound (left panel) or m^1^A antibody-bound poly(A) RNA. Known identities of detected spots are marked. Expected positions of potential m^1^A or m^1^A_m_-initiating nucleotides are marked (blue dashed circles) based on standard migrations in **a**

We readily detected diverse cap dinucleotides using high-resolution liquid chromatography and mass spectrometry using positive ion mode detection. We developed a multiple reaction monitoring protocol based on the fragment ion transitions from distinct dinucleotide precursor species (see Methods). To confirm that the *N*1-methylated adenosine in a cap dinucleotide can be detected, we used synthetic RNA standards (see Methods). Using these standards, we readily detected m^7^G-ppp-m^1^A as well as other cap dinucleotides, such as m^7^G-ppp-A_m_, and m^7^G-ppp-m^6^A_m_ (Fig. 3a).

We next examined endogenous cap dinucleotides prepared by digesting HEK293T poly(A) RNA. We readily detected m^7^G-ppp-m^6^A_m_ (*m/z* *=* 815.1) and also m^7^G-ppp-A_m_ (*m/z* *=* 801.1), though to a lower degree. m^7^G-ppp-C_m_ (*m/z* *=* 777.1), m^7^G-ppp-G_m_ (*m/z* *=* 817.1), and m^7^G-ppp-U_m_ (*m/z* *=* 778.1) were also detected (Fig. 3b). The identity of each species was confirmed by detection of fragment masses corresponding to 7-methylguanine (*m/z* *=* 166.1) and the base comprising the first nucleotide (*m/z* *=* m^6^A_m_ 150.1, A_m_ 136.1, C_m_ 112.1, G_m_ 152.1, U_m_ 112.1) within the extended cap.

Next, we asked whether either m^7^G-ppp-m^1^A or m^7^G-ppp-m^1^A_m_ is present in mRNA. Their masses (*m/z* *=* 801.1 and 815.1, respectively) are identical to cap dinucleotides containing A_m_ or m^6^A_m_. Moreover, the mass of the fragment produced by the *N*^1^-methylated adenine base (*m/z* *=* 150.1) would be identical to that produced by the m^6^A_m_ cap. However, m^7^G-ppp-m^1^A and m^7^G-ppp-m^1^A_m_ exhibit very distinct retention times from the A_m_ and m^6^A_m_ cap dinucleotides based on our synthetic RNA standards (see Fig. 3a). This allows us to differentiate *N*^1^-methyl- and *N*^6^-methyl-containing adenine. Nevertheless, no *N*^1^-methylated adenine-containing cap dinucleotide was detected in mRNA (Fig. 3b). Thus, m^7^G-ppp-A_m_ or m^7^G-ppp-m^6^A_m_ cap structures were readily detected, while m^7^G-ppp-m^1^A_(m)_ was undetectable.

Since mass spectrometry analysis could not validate m^1^A at the transcription-start nucleotide, we wanted to directly and sensitively determine the transcription-start nucleotide that is enriched by the m^1^A antibody. We used two-dimensional thin layer chromatography (2D-TLC), which can identify and quantify the first encoded nucleotide^29^. In this approach, mRNA is decapped, and the exposed 5′ RNA end is radiolabeled, permitting sensitive detection of the first transcribed nucleotide. The radiolabeled nucleotide species are then resolved using 2D-TLC^29^. Based in the mobility of each species, the transcription-start nucleotide can be determined.

We analyzed the transcription-start nucleotide of poly(A) RNA and poly(A) RNA enriched with the m^1^A antibody by 2D-TLC. We also used synthetic RNA containing m^7^G-ppp-m^1^A and m^7^G-ppp-m^1^A_m_ extended cap structures as standards (see Methods). We first optimized the solvent conditions so that m^1^A and m^1^A_m_ migrated to distinct positions by 2D-TLC (Fig. 3c). In poly(A) RNA, no m^1^A or m^1^A_m_ was detected at transcription-start nucleotides (Fig. 3d). Since these might be rare nucleotides at transcription-start nucleotides, we enriched for transcription start m^1^A or m^1^A_m_-containing mRNAs by immunoprecipitating poly(A) mRNA with the AMA-2 m^1^A antibody before 2D-TLC. Here, we again did not see m^1^A or m^1^A_m_ as the transcription-start nucleotide (Fig. 3d).

Taken together, the mass spectrometry and the TLC data suggest that m^1^A and m^1^A_m_ are not readily detectable at the transcription-start nucleotide, and m^7^G-ppp-m^1^A_m_ does not constitute a novel and prevalent mRNA cap structure as proposed^8^.

### The AMA-2 m^1^A antibody recognizes m^7^G-ppp-A cap structures

At this juncture, we had contradictory results: m^1^A-miCLIP suggested that m^1^A is at the transcription-start nucleotide, but we did not observe m^1^A or m^1^A_m_ at this site by either mass spectrometry or TLC. Therefore, we wondered if the AMA-2 antibody binds the transcription-start region in an m^1^A-independent manner. When we originally characterized the specificity of the antibody, we performed classic competition studies using nucleosides or nucleotides. However, based on the binding properties of the antibody revealed by mapping studies, we considered the possibility that the AMA-2 m^1^A antibody could recognize an epitope comprising the mRNA extended cap.

To test this, we used a dot blot assay to measure binding of the AMA-2 m^1^A antibody to an m^1^A-containing oligonucleotide in the presence of various competitors. We considered performing the dot blot assay using m^7^G-ppp-A immobilized on the membrane. However, this approach could be misleading since we do not know if m^7^G-ppp-A interacts with the membrane in a way that would prevent antibody binding. We therefore used the classic competition approach. In this approach, a m^1^A-containing RNA is immobilized on the membrane and different competitors are added in solution. Competitors that bind the m^1^A antibody will prevent the antibody from binding to m^1^A on the membrane.

**Fig. 4:**
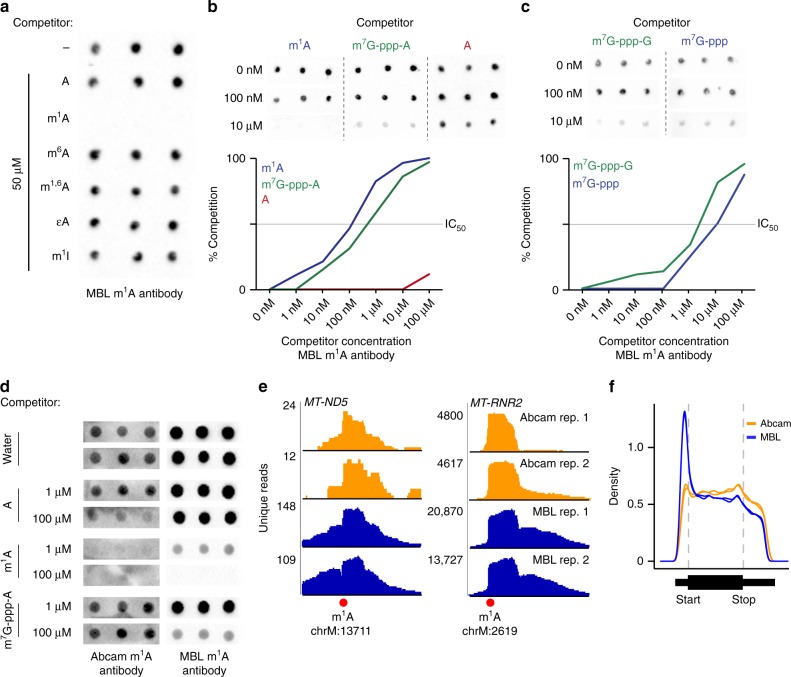
The AMA-2 m^1^A antibody binds to mRNA caps resulting in 5′UTR read enrichment. **a** Shown is a dot blot analysis of the AMA-2 m^1^A antibody’s specificity. An m^1^A-containing oligonucleotide was spotted on a nylon membrane in triplicate and the antibody was incubated with the membrane along with m^1^A-like nucleosides. Only m^1^A prevents antibody binding to the spotted oligonucleotide. m^1,6^A *N*1,6-methyladenosine, εA ethenoadenosine, m^1^I 1-methylinosine. **b** Top, images of dot blot competition assays^1^ where binding of the AMA-2 m^1^A antibody to an m^1^A-containing oligonucleotide was competed with m^1^A (m^1^ATP), m^7^G-ppp-A, or A (ATP) in triplicate. Bottom, quantification of dot blot competition experiments. m^7^G-ppp-A binds (IC_50_ < 1 μM) to the antibody, although the binding is ~10-fold weaker than m^1^A (IC_50_ = ~100 nM). Source data are provided as a Source Data file. **c** The AMA-2 m^1^A antibody recognizes the m^7^G-ppp portion of the cap structure. The membrane was probed with the antibody along with m^7^G-ppp or m^7^G-ppp-G. Both m^7^G-ppp and m^7^G-ppp-G competed with the m^1^A oligonucleotide for binding to the antibody (IC_50_ = ~4 μM for m^7^G-ppp-G, 10 μM for m^7^G-ppp). Source data are provided as a Source Data file. **d** The Abcam m^1^A antibody (ab208196) does not bind the mRNA cap. Dot blots were performed with either of the two m^1^A antibodies (AMA-2 or Ab208196) in the presence of A, m^1^A, or m^7^G-ppp-A (A and m^1^A were either tri- or mono-phosphorylated). While both antibodies bind m^1^A, only the AMA-2 antibody binds m^7^G-ppp-A. **e** The m^1^A antibodies from Abcam (ab208196) and MBL (AMA-2) both detect established m^1^A sites. m^1^A-miCLIP unique reads from two independent antibodies (in replicate) were aligned to the genome, and peaks at known m^1^A sites (red circle) in *MT-ND5* and the mitochondrial 16S rRNA (*MT-RNR2*) exhibited a characteristic truncation at the m^1^A site. **f** The m^1^A antibody Ab208196 lacks the prominent 5′UTR metagene peak seen with the AMA-2 antibody. As in **e**, unique m^1^A-miCLIP reads were aligned to the genome and the relative location of the reads in each transcript is shown with a metagene. Each trace is an independent biological replicate. The 5′UTR peak is essentially absent when the m^1^A-specific Ab208196 antibody was used compared to the cap-cross-reacting AMA-2 antibody

As expected, competition with m^1^A inhibited antibody binding, while related nucleotides, including adenosine, m^6^A, ethenoadenosine, and *N*^1^-substituted nucleotides, like *N*^1,6^-dimethyladenosine (m^1,6^A), did not compete with binding (Fig. 4a). Surprisingly, a commercially available cap analog, m^7^G-ppp-A, was a relatively effective competitor, with an IC_50_ of 480 nM compared to 100 nM for m^1^A (Fig. 4b). m^7^G-ppp-G showed weaker inhibition (IC_50_ ~4 μM) (Fig. 4c). The higher binding to the m^7^G-ppp-A cap analog compared to the m^7^G-ppp-G cap analog may explain the preferential binding of the AMA-2 m^1^A antibody to mRNAs that initiate with adenosine.

Notably, the antibody also showed binding to m^7^G-ppp, but not m^7^G or ATP (Fig. 4c, Supplementary Fig. 4a), suggesting that the antibody’s binding specificity includes recognition of features all along the entire m^7^G-ppp-A extended cap structure.

Since the AMA-2 m^1^A antibody binds the cap structure in an m^1^A-independent manner, the m^1^A peaks seen in the 5′UTR likely reflect binding to the mRNA cap. This would explain why our m^1^A-miCLIP shows read enrichment at the transcription-start nucleotide, as has also been seen using other m^1^A mapping approaches^8^.

To test this hypothesis further, we used a second commercially available m^1^A antibody from Abcam (catalog number ab208196). Unlike the AMA-2 m^1^A monoclonal antibody available from MBL, the Abcam antibody did not bind the m^7^G-ppp-A cap analog (Fig. 4d), indicating that it does not exhibit cross-reactivity with the mRNA cap.

We first confirmed by m^1^A-miCLIP using HEK293T poly(A) RNA that both ab208196 and AMA-2 antibodies can detect authentic m^1^A sites. In each case, we observed a robust peak at the m^1^A sites in *MT-ND5* and *MT-RNR2*, the mitochondrially encoded *16S* RNA (Fig. 4e), confirming the ability of both antibody to detect validated m^1^A sites in mRNA.

We next asked if m^1^A-miCLIP performed using the m^1^A-specific Ab208196 would produce the same transcriptome-wide 5′UTR enrichment of m^1^A-containing fragments observed with the AMA-2 m^1^A antibody^3,4^. As expected, the metagene of the miCLIP fragments using the AMA-2 antibody showed a prominent 5′UTR enrichment (Fig. 4f). However, a metagene analysis of all the immunoprecipitated reads using ab208196 lacked the 5′UTR enrichment (Fig. 4f). Together, these data demonstrate that only the AMA-2 antibody, which cross-reacts with the mRNA cap, results in a 5′UTR enrichment in read coverage. Overall, these data demonstrate that binding to the 5′UTR regions is not linked to the presence of m^1^A at these sites, but rather attributable to cross-reactivity.

### Comparison of m^1^A-miCLIP with earlier m^1^A maps

Although cross-reactivity with mRNA cap structures explains why m^1^A was mapped to transcription-start nucleotides using the AMA-2 antibody, it does not explain the localization of m^1^A to internal sites within the 5′UTR, such as the start codon-proximal region, which was proposed to mediate a novel form of translation initiation^3^. We therefore wanted to understand the exact location of these putative 5′UTR- and start codon-associated m^1^A sites.

When we compared mRNAs that show AMA-2 m^1^A antibody miCLIP coverage with the Dominissini et al.^3^ m^1^A map that localized m^1^A to start codons, we found considerable overlap (Supplementary Fig. 6a). However, the location of reads was different (Supplementary Fig. 6b). In particular, the 5′ ends of the miCLIP reads approached the transcription-start site, while reads from Dominissini et al.^3^ were located downstream of the transcription-start site (Supplementary Fig. 6b, insets). This lateral displacement of peaks towards the start codon is consistent with the library cloning method used in this earlier method (see Supplementary Fig. 1a).

In earlier m^1^A mapping studies, accumulations of reads, or in some cases, “troughs” of reduced read coverage due to a putative m^1^A site, were used to predict m^1^A residues to start codons in mRNA^3^. miCLIP provides more precise positioning by detecting exact sites of antibody-induced crosslinks, rather than using peaks and troughs, which are a common nonspecific feature in RNA-seq data (see Fig. 2b, e and Supplementary Figs. 5c and 6b).

We additionally re-examined the Li et al.^8^ high-resolution m^1^A mapping dataset in HEK293T cells. This study identified 474 m^1^A sites in nuclear-encoded genes based on m^1^A-induced reverse transcriptase misincorporations^8^. However, we eliminated 122 sites for the following reasons: three sites had gene identifiers missing or removed from Refseq, 37 sites did not map to adenosines, and 82 sites were duplicates resulting from mapping to transcript isoforms of the same gene. This left 352 unique putative m^1^A sites in nuclear-encoded genes (see Methods and Supplementary Fig. 6c).

The Li et al.^8^ study located m^1^A sites mostly within the 5′UTR. Only 19 sites were at annotated transcriptional-start sites. However, mRNAs can have alternative transcription-start sites, which differ from the RefSeq-annotated transcription-start site^30^. To determine if the Li et al.^8^ m^1^A sites mapped to alternative transcription-start sites, we compared the reported m^1^A sites to a list of experimentally validated transcription-start sites in HEK293T cells. This list was derived from CAGE-seq and m^6^A_m_ mapping data^31,32^. Of the putative 352 m^1^A sites, 134 overlapped with CAGE and/or m^6^A_m_-inferred transcription-start sites (Supplementary Fig. 6c). Hence, 140 m^1^A sites occurred at transcription-start sites. The false-positive rates of m^1^A mapping is not known, so it is possible that other 5′UTR m^1^A sites are either false positives or map to currently unannotated transcription-start sites. Thus, most, if not all, of the putative start codon/5′UTR m^1^A sites mapped by that study are localized to alternative transcription-start sites, consistent with our mapping results and consistent with the cap-binding properties of the m^1^A antibody.

## Discussion

Considerable attention has revolved around m^1^A based on its description as a high-stoichiometry, translation-promoting modification in thousands of mRNAs located near start codons^3^. Subsequent studies concluded that m^1^A is less prevalent (~700 sites) with a fraction in mitochondrial mRNA^8^, while other studies suggest even fewer sites, with only two mRNAs having an m^1^A at a stoichiometry above 5%^7^. To address these discrepancies, we developed two m^1^A mapping approaches: (1) misincorporation mapping, a computational approach to discover m^1^A-induced misincorporations in ultra-deep RNA-Seq datasets; and (2) m^1^A-miCLIP, a high-resolution method for mapping m^1^A antibody-binding sites in the transcriptome using two different m^1^A-binding antibodies. Misincorporation mapping shows that m^1^A is present at detectable stoichiometries only in the *MT-ND5* transcript, with no m^1^A in other mitochondrial mRNAs or 5′UTRs of mRNAs as reported previously. Using m^1^A-miCLIP, we find that the previously observed binding of the AMA-2 m^1^A antibody to transcription-start nucleotides and the vicinity of start codons is due to a previously unrecognized cross-reactivity of the AMA-2 m^1^A antibody to cap structures. We confirm this using a separate m^1^A antibody that lacks this cap-binding cross-reactivity. We further show that m^1^A is not detectable at transcription-start nucleotides, as previously proposed^8^, based on mass spectrometry and TLC. Overall, these data show that the divergent m^1^A mapping data and large number of 5′UTR-mapped m^1^A sites largely reflect cross-reactivity of the m^1^A antibody with mRNA caps.

Both m^1^A and m^7^G are positively charged purines. This common structural feature may be recognized by the AMA-2 antibody. Since the Ab208196 binds m^1^A but not cap structures, this antibody does not generate 5′UTR false-positive signals. m^1^A-miCLIP carried out using the Abcam antibody shows an m^1^A signature in *MT-ND5* but no transcriptome-wide enrichment in 5′UTRs. These data, along with mass spectrometry, TLC, and misincorporation mapping data, support the idea that the m^1^A localization to 5′UTR sites is specific to the AMA-2 antibody rather than a reflection of bona fide m^1^A nucleotides at these sites.

Our results thus support the idea that m^1^A is a rare and low stoichiometry modification except in the case of *MT-ND5* mRNA, as seen in another study^7^. We were not able to detect m^1^A in any other cytosolic mRNAs or the mitochondrial mRNAs that were reported to contain m^1^A^8^. This could reflect the inability of our method to detect very low stoichiometry m^1^A modifications.

The initial m^1^A mapping studies found that m^1^A is highly prevalent based on mass spectrometry analysis of mRNA^3,4^. However, more recent experiments showed that poly(A) preparations used for mass spectrometry are usually contaminated with tRNA and rRNA, and when these contaminants are meticulously removed, the m^1^A signal in poly(A) preparations is absent^33^. Thus, the newer mass spectrometry data also support the idea that m^1^A is rare.

A pressing question is whether there are more yet-to-be-discovered modified nucleotides in mRNA. Misincorporation mapping suggests that this is not the case, at least for hard-stop nucleotides. The relative paucity of hard-stop nucleotides in mRNA may reflect the incompatibility of these nucleotides with translation. The ribosome mRNA surveillance pathway induces degradation of mRNAs when tRNAs cannot basepair with codons^34^. It is therefore notable that the original m^1^A mapping studies localized many m^1^A sites to coding sequences, which contributed to skepticism about these maps. If hard-stop nucleotides occur in mRNA, they would likely be transient and act to induce mRNA degradation though this surveillance pathway.

## Methods

### Cell lines and animals

For misincorporation mapping, an ultra-deep RNA-seq dataset that profiled RNA expression in blood mononucleocytes was used^12^. For m^1^A-miCLIP, HEK293T cells (passage 5–10, ATCC CRL-3216) or whole mouse brain (16 week age, pooled male and female brain, C57BL/6) was used. HEK293T cells were purchased directly from ATCC but not further validated for identity or tested for mycoplasma contamination. Experiments involving the use of animals were approved by the Institutional Animal Care and Use Committee at Weill Cornell Medicine.

### Antibodies

The AMA-2 m^1^A antibody is a mouse monoclonal antibody (MBL catalog number D345-3). This antibody is documented to react with both m^1^A within RNA and the *N*^1^-methylated adenine base, as documented in MBL’s product specifications for AMA-2 (catalog no. D345-3) and prior studies^3,4^. The specificity was validated previously^3,4^ and in the current study. The Abcam m^1^A antibody (ab208196) is a rabbit monoclonal generated against m^1^A. Its specificity for m^1^A was validated in this study.

### Alignment of reads for misincorporation mapping

Raw reads from the ultra-deep RNA-seq dataset used for this study^12^ were downloaded from GEO (accession code: GSE33029). This RNA-seq dataset was prepared using standard reverse transcription with SuperScript III, an enzyme expected to produce misincorporations at m^1^A positions^11^. Variants identified in the genomic DNA corresponding to this dataset were acquired from http://snyderome.stanford.edu. Coordinates of other SNPs that may be present in the DNA sequence were downloaded from the SNP database dbSNP (February 2017 build; https://www.ncbi.nlm.nih.gov/projects/SNP/). Read alignment of forward and reverse read mates was performed using STAR (version 2.5.3a) and the hg19 genome build. Alignment incorporated removal of PCR duplicates, and clipping of 10 bases on either end of each read, since the ends of Illumina reads are prone to sequencing error^35^. Only reads that mapped to a single location in the genome were used for downstream analysis. A maximum of one mismatch per read was permitted for alignment.

### Misincorporation mapping

To identify misincorporations, aligned reads were analyzed using Rsamtools Pileup (version 1.27.16). This program was used to determine the frequency of each of the four nucleotides present in mapped reads at every genomic position with read coverage. We limited our analysis to nucleotide positions with a minimum combined read depth of 500 unique reads across the 20 biological replicates to maximize sensitivity of detecting modified nucleotides. To prevent calling genomic variants and SNPs as modification-induced misincorporations, we did not analyze nucleotide positions containing variants discovered in the genomic DNA corresponding to the RNA-seq dataset, or SNPs annotated in dbSNP. Importantly, our analysis could only be performed on transcripts longer than the library insert size of ~250 bases^12^. For this reason, analysis of cytosolic tRNAs, which are ~75 nt-long RNAs that contain known conserved m^1^A residues, could not be performed. However, short RNAs generated from polycistronic transcripts, like mitochondrial tRNAs^36^, were represented in the analyzed library. To identify sites of modification throughout the transcriptome, we initially filtered for all nucleotide positions that were covered by at least 500 mapped reads and contained a 1% misincorporation rate, and that were present in at least half of biological replicates (Supplementary Data 1). To further obtain a high-confidence list of modification positions, we required that within the misincorporation profile at each initially identified position, a minimum of 5% of misincorporations were heterogeneous (i.e. transitions of the reference nucleotide to all three possible alternative nucleotides) in order to minimize detection of adenosine-to-inosine editing, and heterozygous alleles not reported as variants or SNPs. We chose this filter because hard-stop nucleotides have been shown to cause heterogeneous misincorporations, even when one type of misincorporation is predominant^11^. This resulted in a high-confidence list of sites that were detected at known and novel modification positions (Supplementary Data 2).

### m^1^A-miCLIP

m^1^A-miCLIP was performed as previously described^20,37^, briefly described below along with any modifications: C57BL/6 mice (8 weeks) were sacrificed by CO_2_ inhalation and cervical dislocation as approved by the Weill Cornell Medicine Institutional Animal Care and Use Committee (IACUC). Total RNA from HEK293T cells (*n* = 2 biological replicates) or whole mouse brain (*n* = 6 biological replicates) was extracted using TRIzol (ThermoFisher) and treated with RNase-free DNase I (Promega). Poly(A) RNA was isolated using one round of selection with oligo(dT)_25_ magnetic beads (New England Biolabs). This resulted in approximately 10 μg of poly(A) RNA for each replicate used in this study. Poly(A) RNA was subjected to fragmentation using RNA Fragmentation Reagents (ThermoFisher) for exactly 12 min at 75 °C. This fragmentation protocol is identical to the one in m^1^A-seq, and has been reported not to facilitate substantial m^1^A to m^6^A rearrangement.^3^ Fragmented RNA was then incubated with 10–15 μg of m^1^A antibody per replicate and the antibody-RNA complexes were processed for crosslinking, immunoprecipitation, RNA 3′ linker ligation, purification, and reverse transcription^20,37^. Following reverse transcription of purified peptide-RNA complexes, first-strand cDNA was circularized using CircLigase II ssDNA Ligase (EpiBio) to preserve the 3′ end of the cDNA, and thus, sites of m^1^A-induced terminations of reverse transcription. To generate priming sites for library amplification, the cDNA was cut in the middle of the cDNA primer sequence using a single-stranded DNA oligo complementary to this sequence and FastDigest *Bam*HI (ThermoFisher)^20,37^. This generated priming sites for the Illumina P5 and P3 primers on either side of the first-strand cDNA, eliminating the need for a second-strand synthesis step. For library amplification, Accuprime Supermix I (ThermoFisher) and Illumina P5 and P3 primers were used (see Supplementary Data 7). Amplified libraries were purified using AMPure XP magnetic beads (Beckman Coulter). Libraries were subjected to next-generation sequencing at the Epigenomics Core of Weill Cornell Medicine. Libraries were sequenced on an Illumina HiSeq 2500 and MiSeq instrument in single-end mode to generate 50-base reads.

### RNA-seq

HEK293T cell total RNA was extracted with TRIzol (ThermoFisher), treated with RNase-free DNase I (Promega), and poly(A) RNA was isolated using one round of selection with oligo(dT)_25_ magnetic beads (New England Biolabs). RNA was then subjected to fragmentation using RNA Fragmentation Reagents (ThermoFisher) for exactly 12 min at 75 °C. Fragmented RNA was then subjected to RNA 3′ linker ligation using T4 RNA Ligase I (New England Biolabs) and reverse transcription using a primer complementary to the linker sequence and SuperScript III (ThermoFisher) (see Supplementary Data 7). First-strand cDNA was gel-purified using denaturing PAGE, and then circularized using Circligase II ssDNA Ligase (EpiBio). Circularized cDNA was then cut and amplified exactly as described above for m^1^A-miCLIP. Resulting RNA-seq libraries were subjected to next-generation sequencing at the Epigenomics Core of Weill Cornell Medicine. Libraries were sequenced on an Illumina HiSeq 2500 instrument in single-end mode to generate 50-base reads.

### Read processing and alignment

After sequencing, reads from m^1^A-miCLIP or RNA-seq libraries were trimmed of the 3′ linker sequence and barcoded reverse transcription primer sequences using Flexbar (version 2.5) (see Supplementary Data 7). To demultiplex reads belonging to individual biological replicates, the pyBarcodeFilter.py script of the pyCRAC suite (version 1.2.2) was used. The random portion of the reverse transcription barcode was then moved into the sequence header using a custom awk script (available upon request). PCR duplicates were collapsed using pyFastqDuplicateRemover.py of the pyCRAC suite. Finally, reads were aligned to hg19 for HEK293T cells or mm10 for mouse brain using Bowtie (version 1.1.2).

### Generation of m^1^A-miCLIP clusters

m^1^A-miCLIP clusters of unique reads were generated using the CIMS software package for analysis of HITS-CLIP data^38,39^. To generate clusters and determine the cluster score (maximum of stacked reads), the tag2profile.pl, tag2cluster.pl and extractPeak.pl scripts of the CIMS software package were used. A custom awk script was then used to filter for clusters of a minimum score (at least 20 stacked reads; script is available upon request).

### Motif analyses

To search for a possible common sequence motif present in our HEK293T cell m^1^A-miCLIP dataset, we focused on potential motifs present in m^1^A-miCLIP clusters in the 5′UTR, the region of predominant m^1^A-miCLIP cluster enrichment. The genomic sequences of these clusters were retrieved using bedtools and subjected to motif discovery using the MEME suite (version 4.11.4).

### Metagene distribution analyses

To analyze the metagene distribution of m^1^A-miCLIP clusters on mRNAs, MetaPlotR was used^40^, with in-house modifications. The density of m^1^A-miCLIP coverage was normalized to that of RNA-seq coverage to reveal any enrichments using a custom R script (available upon request). For the HEK293T cell metagene, the in-house HEK293T cell RNA-seq dataset described above was used for normalization. For the mouse brain metagene, a published whole-brain RNA-seq dataset was used^41^ (accession code: GSE52564). To plot the coverage of transcription-start sites by m^1^A-miCLIP at higher resolution, the plotProfile tool of the Deeptools suite was used.

### Examination of antibody crosslink sites around the transcription-start site

For analysis of antibody crosslinks at the transcription-start sites of mRNAs, we analyzed terminations of reverse transcription (i.e. 5′ ends of reads) around these sites. To do so, the number of terminations was measured around RefSeq-annotated transcription-start sites that had coverage in both m^1^A-miCLIP and RNA-seq. Terminations were counted at positions ranging from the transcription-start site to position +4 relative to the transcription-start site. Then, transcription-start sites were filtered for those that contained a minimum coverage of five unique reads at the transcription-start site position in both m^1^A-miCLIP and RNA-seq. This filtered set of transcription-start sites was then used to compare the distributions of read terminations in m^1^A-miCLIP and RNA-seq. We focused on terminations rather than misincorporations in m^1^A-miCLIP is because the misincorporation profile of m^1^A is sequence dependent, with both upstream and downstream nucleotides contributing to misincorporation variability^11^. Thus, we used the presence of terminations as a signature of antibody crosslinking events in our dataset. Additionally, while rare types of reverse transcriptases that read through m^1^A have been described^42^, standard reverse transcriptases, like the SuperScript III used in m^1^A-miCLIP, produce frequent terminations at m^1^A residues^11,43^.

### Measurement of transcription-start sites enriched by m^1^A-miCLIP

To determine the types of transcription-start sites overlapping m^1^A-miCLIP clusters, we used a collection of transcription-start sites that included RefSeq transcription-start sites as well as recently-mapped transcription-start site regions containing the m^6^A_m_ mRNA extended cap^24^. The frequencies of all transcription-start site types or those overlapping m^1^A-miCLIP clusters were thus determined using this collective set.

### Synthetic oligonucleotides used in this study

For biochemical analysis of various modifications present within the extended caps of mRNAs, synthetic oligonucleotides were generated as standards for mass spectrometry and/or thin layer chromatography (see below; see Supplementary Data 7). Oligonucleotides containing m^7^G-ppp-A_m_, m^7^G-ppp-m^6^A, or m^7^G-ppp-m^6^A_m_ were synthesized chemically^44^. Oligonucleotides containing m^7^G-ppp-m^1^A or m^7^G-ppp-m^1^A_m_ were synthesized enzymatically using an oligonucleotide initiating with ppp-m^1^A (Trilink). This oligonucleotide was capped using ScriptCap Cap 1 Capping System (CellScript) to generate the m^7^G cap and, in the case of m^7^G-ppp-m^1^A_m_, 2′-*O*-methylation of m^1^A.

### Liquid chromatography and mass spectrometry (LC-MS)

Poly(A) RNA was prepared for mass spectrometry as follows. Total RNA from HEK293T cells was treated with TURBO DNase (ThermoFisher) according to the manufacturer’s instructions, followed by two rounds of poly(A) selection using oligo(dT) magnetic beads (NEB). Small RNAs shorter than 200 nt were then removed from the poly(A) RNA using the RNeasy kit (Qiagen). This size selection was performed to prevent detection of extended cap structures that are known to be present in certain small RNAs, like small nuclear RNAs (snRNAs). Approximately 5 μg of DNase-treated, poly(A)-selected, and size-selected RNA was thus generated for each sample for mass spectrometry analysis. To release extended cap structures from the nucleotides comprising the internal portion of the RNA, RNA was digested with 2–4 units of Nuclease P1 (Sigma Aldrich) in a final buffer concentration of 30 mM sodium acetate (pH 5.5) for 3 h at 37 °C. Following digestion, the nuclease was removed from the samples using molecular weight cutoff centrifugal filters (VWR). The digested and purified RNA was finally dried using an Eppendorf Vacufuge and reconstituted with 70% acetonitrile (LC-MS grade; Sigma Aldrich) to a final concentration of 0.5 µg/µl. Two microliters of the resulting solution were subjected to MS analysis.

Samples were injected into an LC-MS/MS system comprising an Agilent 1260 HPLC and an Agilent 6460 triple quadrupole mass spectrometer equipped with a JetStream electrospray ionization source. Positive ion monitoring and multiple reaction monitoring was used for detection of extended caps. The caps were resolved on an aqueous normal phase column (ANP, Cogent Diamond Hydride, 4 µm particle size, 150 mm × 2.1 mm; Microsolv). To achieve chromatographic separation of the cap structures from mononucleotides, the following gradient was used. The aqueous mobile phase (Buffer A) was 50% isopropanol with 0.025% acetic acid, and the organic mobile phase (Buffer B) was 90% acetonitrile containing 5 mM ammonium acetate. EDTA was added to the mobile phase in a final concentration of 6 µM. The final gradient applied was 0–1.0 min 99% B, 1.0–7.0 min to 80% B, 7.0–18.0 min to 50% B, 18.0–19.0 min to 0% B, and 19.1–29.0 min 99% B. The flow rate was 0.4 mL/min during data acquisition and 0.6 mL/min during column re-equilibration. Data were saved in centroid mode using MassHunter workstation acquisition software (Agilent). Data files were processed with MassHunter Qualitative Analysis Software (Agilent).

Exact operating source parameters for the LC-MS analysis are available upon request.

### Biochemical examination of modifications at transcription-start sites

To identify the initiating nucleotide structure in mRNAs bound by the m^1^A antibody, we utilized 2D-TLC analysis of mRNA extended caps^24^. For analysis of cellular RNA, we used HEK293T cell poly(A) RNA or poly(A) RNA enriched using the m^1^A antibody^3,4^. Oligonucleotide standards, input (antibody-unbound) poly(A) RNA, and m^1^A antibody-enriched poly(A) RNA were then subjected to 2D-TLC^24^. To enhance resolution of m^1^A and m^1^A_m_ from other nucleotide species, the first dimension of 2D-TLC was resolved using 66% isobutyric acid and 1% NH_4_OH in water, and the second dimension was resolved using 60% ammonium sulfate in 100 mM sodium phosphate buffer of pH 6.8 (w/v) with a final concentration of 2% *n*-propanol. Both dimensions were resolved overnight.

### Synthesis of *N*^1,6^-methyladenosine

To synthesize *N*^1,6^-methyladenosine, *N*^6^-methyladenosine (Selleckchem) was dissolved in dry DMF and followed with addition of iodomethane (Acros Organics; 10:1 molar ratio iodomethane:*N*^6^-methyladenosine). The mixture was stirred overnight at room temperature. The product was purified by flash chromatography on silica gel (EMD), eluting with methanol and dichloromethane (1:10 to 1:5; ACS or HPLC grade solvents). This resulted in a product yield of 46.3% *N*^1,6^-methyladenosine. Product identity was confirmed by nuclear magnetic resonance (NMR) and high-resolution mass spectrometry (HR-MS).

NMR spectra were recorded using a 500-MHz Bruker DMX-500 instrument at room temperature, and chemical shifts were referenced to the residual solvent peak. Shifts were as follows: ^1^H NMR (500 MHz, DMSO-*d*_6_) δ 8.11 (s, 1H), 8.04 (s, 1H), 5.76 (d, *J* = 5.8 Hz, 1H), 5.15 (s, 1H), 4.43 (t, *J* = 5.4 Hz, 1H), 4.15 (m, 2H), 3.92 (d, *J* = 3.7 Hz, 1H), 3.63 (dd, *J* = 12.0, 3.9 Hz, 1H), 3.56 (m, 2H), 3.50 (s, 3H), 3.45 (d, *J* = 6.5 Hz, 1H), 1.23 (s, 3H).

HR-MS data were recorded with Waters LCT-Premier XE at room temperature. For a predicted mass for *N*^1,6^-methyladenosine, or C_12_H_18_N_5_O_4_^+^, of 296.1353, the mass found was 296.1361.

### Analysis of Li et al. m^1^A sites

The Li et al.^8^ m^1^A sites for nuclear-encoded genes was obtained from supplementary Table 2 of the published manuscript. We annotated each of the 474 transcriptomic sites with their corresponding genomic coordinates and nucleotide sequences using an annotation file generated from Refseq with MetaPlotR. With a custom R script, we then filtered likely erroneous sites as specified in the Results section. Briefly, sites corresponding to gene IDs missing in Refseq, or that mapped to non-adenosine nucleotides, or with duplicate genomic coordinates were all removed.

### Characterization of antibody affinity for various substrates

The specificity or affinity of the m^1^A antibody for various nucleosides, nucleotides, or cap structures was determined as follows. To determine the specificity of the m^1^A antibody for various nucleosides, two approaches were performed. For testing the specificity of the antibody for various nucleosides in the context of m^1^A-miCLIP, the antibody was crosslinked to total cellular RNA in the presence of various competitor nucleotides. Antibody binding, crosslinking, and detection of crosslinked antibody-RNA complexes was performed exactly as in miCLIP, except with the inclusion of the competitor nucleotide during the antibody-binding reaction.

For testing the specificity of the antibody for modified adenines, especially those resembling *N*^1^-methylated adenine, a dot blot assay was performed wherein the competing molecule is added during antibody binding^1^. Competition assays are usually used to measure binding, rather than spotting the nucleotides to the membrane, since the manner of interaction of each nucleotide to the membrane is not known and can affect antibody binding. For measuring the affinity, the IC_50_ of the various nucleotides and cap dinucleotides was measured. In these experiments, a series of the dot blot assays were performed, where serial dilutions of each competitor molecule (ranging from 10 µM to 1 nM) were used in parallel during antibody binding reactions. The dot blots were performed as follows: 250 ng of m^1^A-containing synthetic oligonucleotide (see Supplementary Data 7) were spotted in triplicate on a BrightStar membrane (ThermoFisher), allowed to briefly air-dry, and auto-crosslinked twice in a Stratalinker 2400 (Stratagene). Each membrane was rinsed briefly in PBST, then blocked for 1 h at room temperature in 5% milk in PBST. Each membrane was then placed into a pouch containing a 1:1000 dilution of the m^1^A antibody in 0.5% milk in PBST, and an appropriate concentration of competitor molecule. The antibody binding proceeded for 2 h at room temperature. Then, each membrane was washed three times in PBST (5 min per wash), and then incubated in a dilution of 1:2500 of secondary antibody (anti-mouse, GE # NA931; anti-rabbit, GE NA934) in 0.5% milk in PBST for 1 h at room temperature. Finally, the membrane was washed three times in PBST (5 min per wash), and developed using ECL Prime (GE). Membranes corresponding to a dilution series of a specific competitor molecule were imaged together using a ChemiDoc Imager (Bio-Rad).

### Reporting summary

Further information on research design is available in the Nature Research Reporting Summary linked to this article.

## Supplementary information

Supplementary Information

Reporting Summary

Description of Additional Supplementary Files

Supplementary Data 1-7

Source Data

## Data Availability

A reporting summary for this Article is available as a Supplementary Information file. Sequencing data have been deposited in GEO (GSE97909). The source data for Figs. 2e, 4b and 4c are provided in the Source Data File. All data are available from the corresponding author upon reasonable request.

## Code availability

Custom scripts used in this study are available here: https://github.com/olarerin/misincorporation_mapping.
