# Supplementary material for: Antibody cross-reactivity accounts for widespread appearance of m1A in 5’UTRs
Source: Nat Commun. 2019 Nov 12;10:5126. doi: 10.1038/s41467-019-13146-w (PMC6851129; doi:10.1038/s41467-019-13146-w)
Supplement: Supplementary file 1 — Supplementary Information [file 41467_2019_13146_MOESM1_ESM.pdf]

Antibody cross-reactivity accounts for widespread appearance of m^1A in 5'UTRs

Grozhik et al.

Supplementary Information

Grozhik et al. Supplementary Figure 1

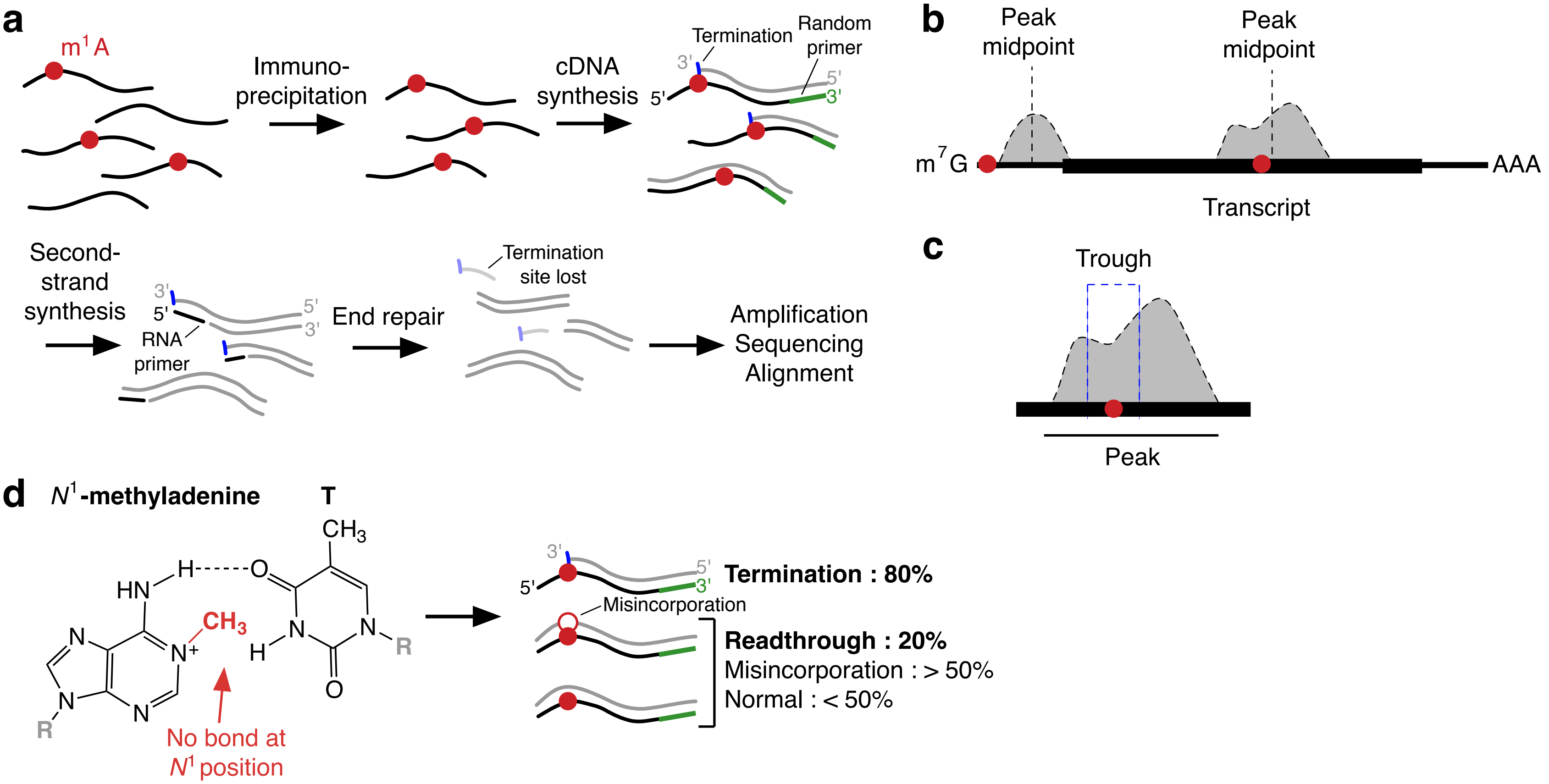

Supplementary Figure 1. m^1A mapping by immunoprecipitation and its effect on base-pairing

a, Earlier strategy for mapping m^1A. Earlier transcriptome-wide m^1A mapping approaches[3,4] generated sequencing libraries from RNA fragments containing m^1A. To achieve this, RNA (black lines) was fragmented, immunoprecipitated using a m^1A antibody, and reverse transcribed using primers (green bars). m^1A (red circles) predominantly causes termination of standard reverse transcription, and only a small fraction of cDNAs (grey lines) go through the m^1A residue. Although the termination site marks the position of the m^1A, the exact site of the reverse transcription termination is not preserved in these methods. This is because during cloning of the cDNA library, the second-strand cDNA is generated in a manner that uses RNase-H to nick the RNA template. This RNAse-H-generated nick produces an RNA primer (short black line) for second-strand synthesis. Following second-strand synthesis, the RNA primer and the 3' end of the first-strand cDNA are lost during end repair (i.e. blunting) of double-stranded cDNA ends. Thus, in earlier m^1A mapping approaches, the site where the reverse transcriptase terminates is lost, preventing m^1A residues from being detected at nucleotide resolution.

b, Peaks generated by earlier m^1A mapping approaches can be displaced from the m^1A site. Although the precise sites of reverse transcription terminations are not preserved in earlier m^1A mapping approaches, peaks (grey) were used to infer the presence of m^1A (red circles) in transcripts (black bar). If the m^1A is at an internal site, reads will be generated on either side of the m^1A. The resulting peak is likely to cover the m^1A site (right). The "peak midpoint," which was previously reported for these peaks[3], is likely to be near the m^1A site. A different result happens if the modification is at the 5' end of mRNAs (left). In this case, because cDNA ends are not preserved (see **a**), peaks will be displaced downstream of the m^1A site and the peak midpoint will not reflect the location of the modified residue. Since the cDNA termination site is lost, there will be no way to identify modified sites at the 5' ends of transcripts. In contrast, m^1A-miCLIP can detect either an both internal and 5' terminal modifications of the mRNA since the termination sites are preserved.

c, Peak troughs proposed to enhance m^1A mapping resolution in earlier approaches[3]. If an m^1A is located at an internal site in the mRNA (i.e. not at the transcription-start site), regions containing m^1A residues (red circle) can be narrowed down based on the regions of reduced read coverage, or "troughs," (box) within peaks (grey). Troughs are thought to occur at m^1A residues because cDNA generated by reverse transcription can be primed upstream of the m^1A residue, or downstream of the m^1A residue. However, troughs are a relatively nonspecific feature of RNA-seq and are especially difficult to distinguish from noise in low abundance or low coverage mRNAs.

d, Mechanism of the effect of N^1-methylated adenine on reverse transcription. N^1-methylated adenine leads to termination of reverse transcription or misincorporation of nucleotides because the N^1 methyl on the adenine base blocks formation of a hydrogen bond that is necessary to pair with thymine. As a result, N^1-methylated adenine and m^1A cause termination of reverse transcription, and any readthrough of reverse transcriptase through this modification is often accompanied by misincorporation of the complementary nucleotide.

Grozhik et al. Supplementary Figure 2

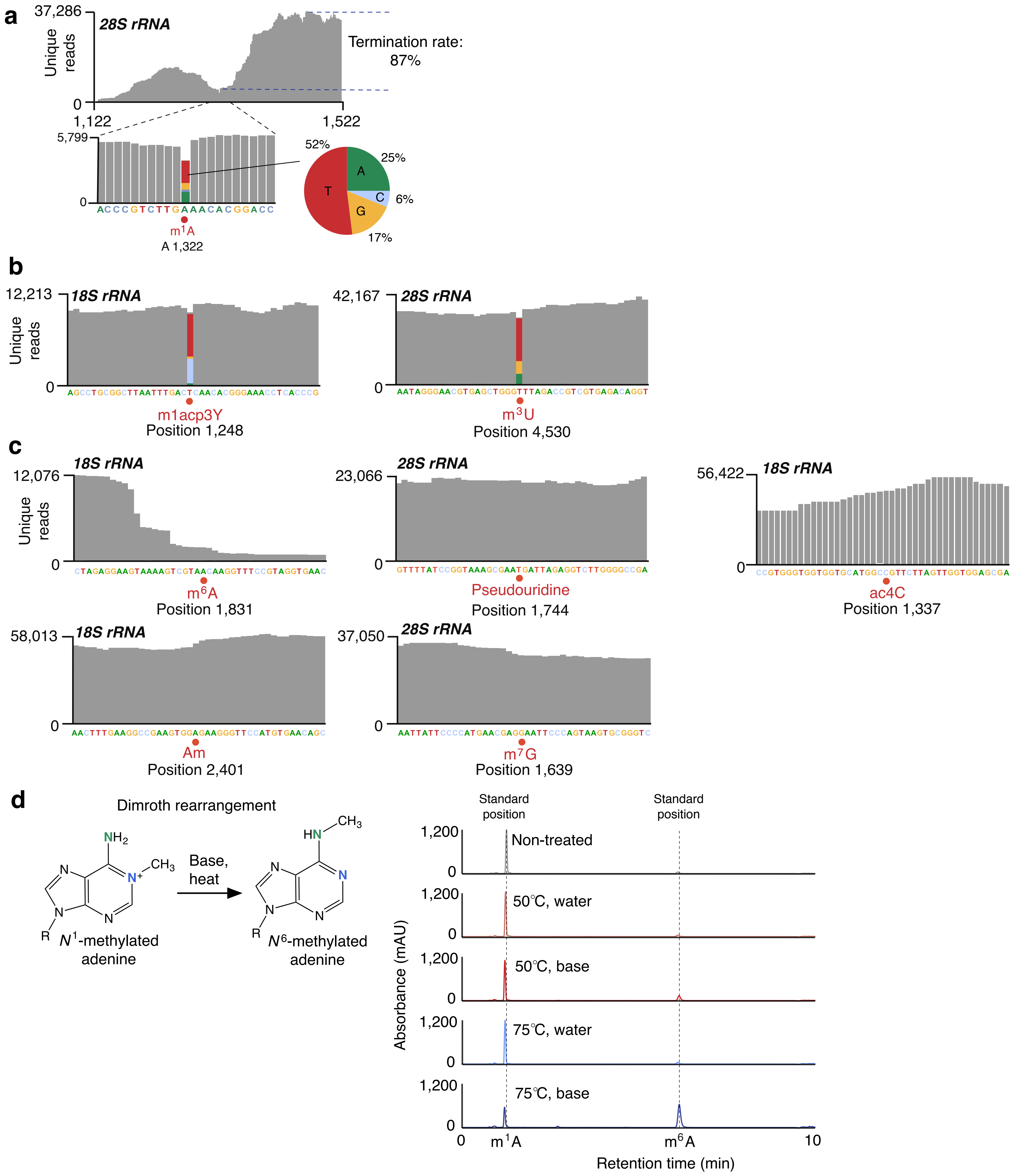

Supplementary Figure 2. Validation of misincorporation mapping

a, Misincorporation mapping detects m^1A in the *28S* rRNA. Shown is the quantification of misincorporations described in **Fig. 1b**. As expected, the m^1A residue at this position was marked by a high rate of misincorporations. 17% of the reads (grey) at this position showed an A→G transition, 6% of reads contained an A→C transition and most reads—52%—contained an A→T transition. Together, this misincorporation profile is characteristic of the reverse transcription signature of m^1A[9]. The detection of misincorporations was important for our study because the RNA-seq dataset we used for misincorporation mapping was cloned in a manner that did not preserve m^1A-induced reverse transcription terminations[11]. Thus, we relied on misincorporations to map hard stop modifications like m^1A. This result demonstrates that misincorporation mapping can be used to map m^1A residues using a misincorporation profile that is typical of m^1A.

We considered that in our misincorporation mapping dataset, Dimroth rearrangement could have occurred, preventing the detection of m^1A residues. However, given our ability to identify m^1A in the *28S* rRNA using this strategy, this appears unlikely. To estimate how much m^1A was lost during library preparation, we measured the termination rate seen in the *28S* rRNA. As can be seen, there is high coverage downstream of the m^1A. This drops substantially at the m^1A site, reflecting a read termination rate of ~87%. This is consistent with a near complete preservation of m^1A in this RNA. Thus, we reasoned that Dimroth rearrangement would not substantially impair our ability to detect m^1A in this dataset.

b, Misincorporation mapping detects other hard stop modifications. In addition to detecting the m^1A residue in the *28S* rRNA, misincorporation mapping reliably identified other hard stop modifications in rRNA. In the *18S* rRNA, 1-methyl-3-(3-amino-3-carboxypropyl)pseudouridine (m^1Acp3Y) was marked by reads (grey) containing a high rate of misincorporations. Likewise, another hard stop modification, m^3U, in the *28S* rRNA, was also marked by misincorporations. Thus, misincorporation mapping can identify a variety of hard stop modifications.

c, Misincorporation mapping does not detect modifications that do not affect reverse transcription. To validate that modification mapping specifically detected hard stop modifications, we examined the misincorporation profiles of modifications in rRNA that are known not to affect reverse transcription. As expected, we found that four modifications that do not affect reverse transcription, including m^6A and 2'-*O*-methyladenosine (A_m) in the *18S* rRNA, and pseudouridine and m^7G in the *28S* rRNA, produced no misincorporations in the reads (grey) at their respective positions. Thus, misincorporation mapping is specific to hard stop modifications, and not modifications that do not affect reverse transcription.

d, m^1A is relatively stable under routine laboratory conditions, but unstable in the presence of base and heating. Shown is the m^1A to m^6A conversion via the Dimroth rearrangement (left mechanism). Under basic conditions and high temperatures, m^1A can convert to m^6A (right). Shown is an HPLC assay to detect conversion of m^1A (0.5 mM) to m^6A in the following conditions: 25°C (water, 30 min), 50°C (water, 30 min), 75°C (water, 30 min), 50°C (10 mM sodium bicarbonate, pH 9.0, 30 min), 75°C (10 mM sodium bicarbonate, pH 9.0, 30 min). Minimal conversion was seen except at 75°C in basic conditions. Under these conditions, ~50% conversion was seen. HPLC assay conditions were as previously described[23].

Grozhik et al. Supplementary Figure 3

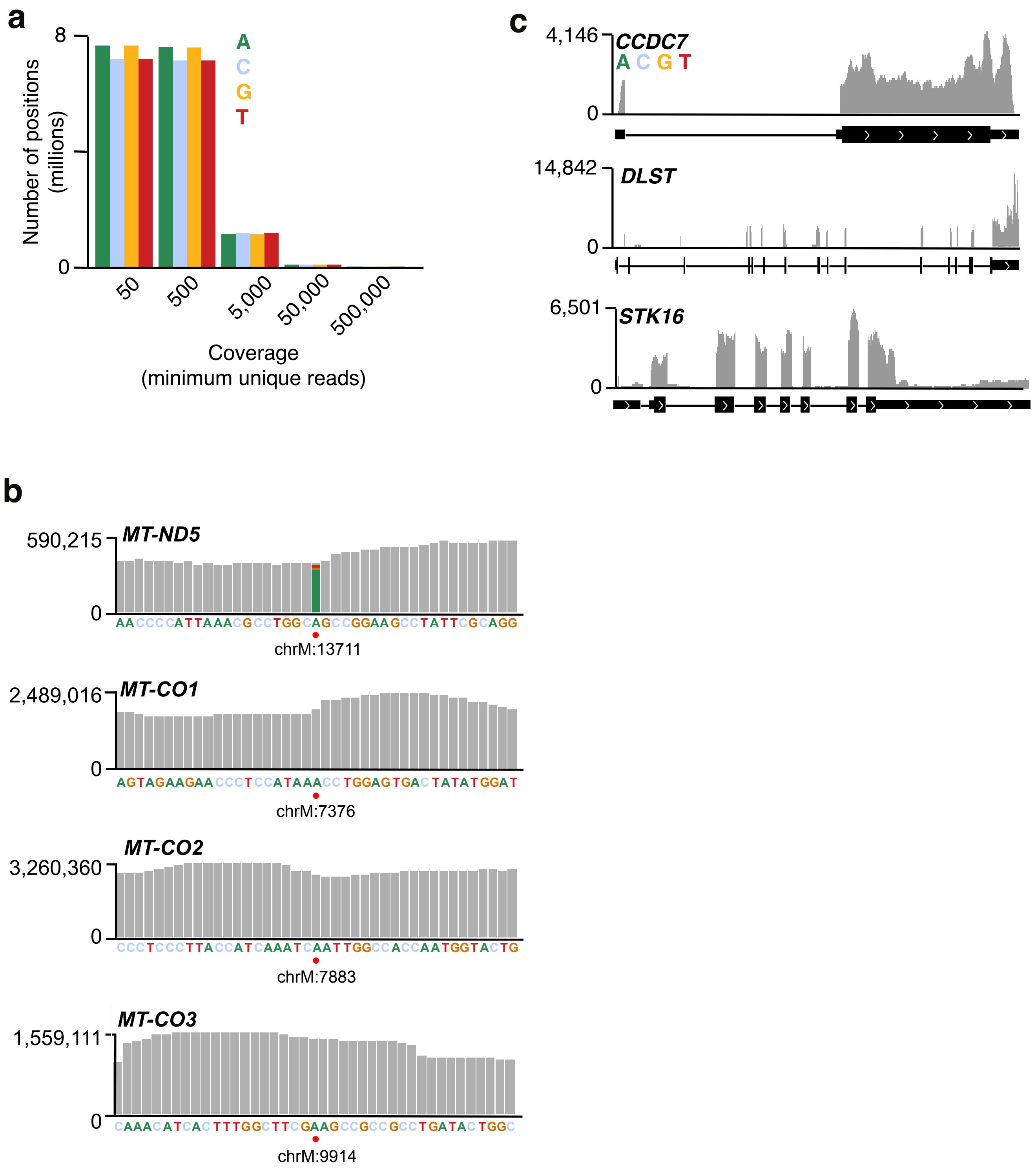

Supplementary Figure 3. Misincorporation mapping in mRNA

a, Selection of read depth criteria for misincorporation mapping. To detect modification-induced misincorporations throughout the transcriptome, we first examined the coverage depth of the ultra-deep RNA-seq dataset at all genomic positions. At positions that had coverage, many positions were covered by 50 to 500 unique reads. However, for our analysis, we chose to analyze the subset of nucleotides that were covered by 500 or more unique reads in order to maximize the sensitivity of detecting hard stop modifications in mRNA. Here, in total, approximately 8 million positions of each type of nucleotide were covered by 500 or more reads. Thus, many nucleotides throughout the transcriptome were able to be evaluated for hard stop modifications.

b, Examination of misincorporations in putative m^1A-containing mitochondrial transcripts. The indicated positions in *MT-ND5*, *MT-CO1*, *MT-CO2* and *MT-CO3* were identified as putative m^1A sites in two previous studies. A robust m^1A misincorporation signature was seen for the *MT-ND5* site but not at the m^1A sites in the other genes. Thus, with the exception of *MT-ND5*, misincorporation mapping indicates that these mRNAs lack high stoichiometry m^1A sites. Of note, the read coverage in all genes was exceptionally high, allowing for sufficient read depth needed to identify low stoichiometry m^1A sites. The exact misincorporation rate at each m^1A site is listed in **Supplementary Table 3** for each putative m^1A-containing mitochondrial mRNA. Position of putative m^1A sites for each transcript is indicated with a red dot.

c, Examination of misincorporations on high-stoichiometry m^1A-containing transcripts. Previously identified m^1A containing transcripts, *CCDC7*, *DLST*, and *STK16*, were shown in an earlier study to contain m^1A residues of greater than 50% stoichiometry. However, no adenosines in these transcripts showed misincorporation profiles that met our filtering criteria (see **Methods**), despite high depth of coverage (black, transcript models; white arrows, direction of transcription). Thus, misincorporation mapping did not detect m^1A residues in the transcripts previously reported to contain m^1A at high stoichiometry.

Grozhik et al. Supplementary Figure 4

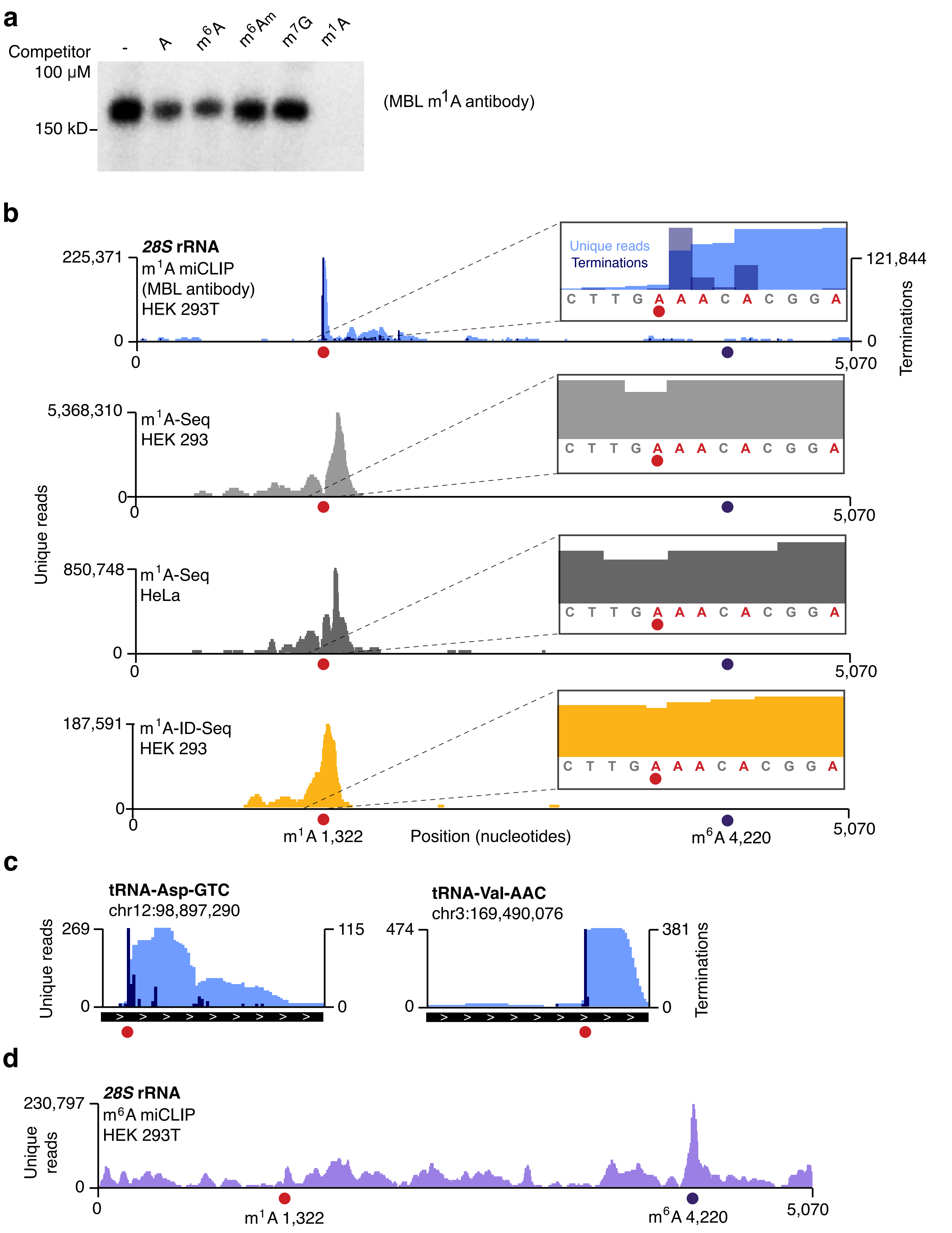

Supplementary Figure 4. m^1A-miCLIP validation with the MBL m^1A antibody

a, The MBL m^1A antibody detects m^1A RNA. Although the specificity of the m^1A antibody was previously validated[3,4], we wanted to validate its specificity in the context of the m^1A-miCLIP protocol. To do this, we incubated the antibody with total fragmented RNA from HEK293T cells in the presence of 100 μM of various competitor nucleotides. Then, the antibody was crosslinked to the RNA and the crosslinked antibody-RNA complexes were radiolabeled and purified by SDS-PAGE and membrane transfer. While some competitor nucleotides weakly inhibited antibody-RNA crosslinking, only m^1A abolished all crosslinking. Moreover, we did not readily detect binding of the antibody to these other nucleotides when the RNA was subjected to sequencing (see **b**).

b, m^1A-miCLIP detects the m^1A residue in the *28S* rRNA. To further assess the specificity of m^1A-miCLIP, we aligned unique reads from m^1A-miCLIP (light blue) or earlier m^1A mapping approaches (m^1A-seq[3], grey; m^1A-ID-seq[4], yellow) to rRNA. Like earlier m^1A mapping approaches, the m^1A-miCLIP protocol resulted a striking accumulation of unique reads at the m^1A residue at position 1,322 (red circle) of the *28S* rRNA. Notably, no enrichment of reads was observed at the m^6A residue at position 4,220 (purple circle), or anywhere else, confirming a lack of cross-reactivity of the antibody to N^6-methylated adenine or other modified nucleotides, which are abundant in rRNA. Moreover, while all protocols resulted in read accumulations at the m^1A residue, the m^1A-miCLIP peak was markedly narrower than the peaks produced by earlier strategies. Furthermore, in m^1A-miCLIP, this m^1A residue was marked by many read terminations (dark blue) at the +1 position of the m^1A residue. Thus, m^1A-miCLIP marks m^1A residues with high specificity and resolution.

c, m^1A-miCLIP detects m^1A in tRNAs. To validate the specificity of m^1A-miCLIP further, we examined tRNAs (black bars; white arrows, direction of transcription), which contain conserved m^1A residues (red circles, MODOMICS annotations[45]). Here, two example tRNAs are shown. Like in the *28S* rRNA, in these tRNAs, m^1A-miCLIP reads (light blue) and terminations (dark blue) marked m^1A residues with high specificity and resolution.

d, miCLIP does not cause rearrangement of m^1A to m^6A. To determine whether the miCLIP protocol may miss detection of m^1A residues due to Dimroth rearrangement of m^1A to m^6A, we examined the presence of potential m^6A at the *28S* rRNA m^1A residue position. While the conserved m^6A residue was marked by a high enrichment of accumulated m^6A-miCLIP reads (purple circle), reads found at the position of the m^1A residue (red circle) were present at background level. This suggests minimal conversion of m^1A to m^6A during the miCLIP protocol.

Grozhik et al. Supplementary Figure 5

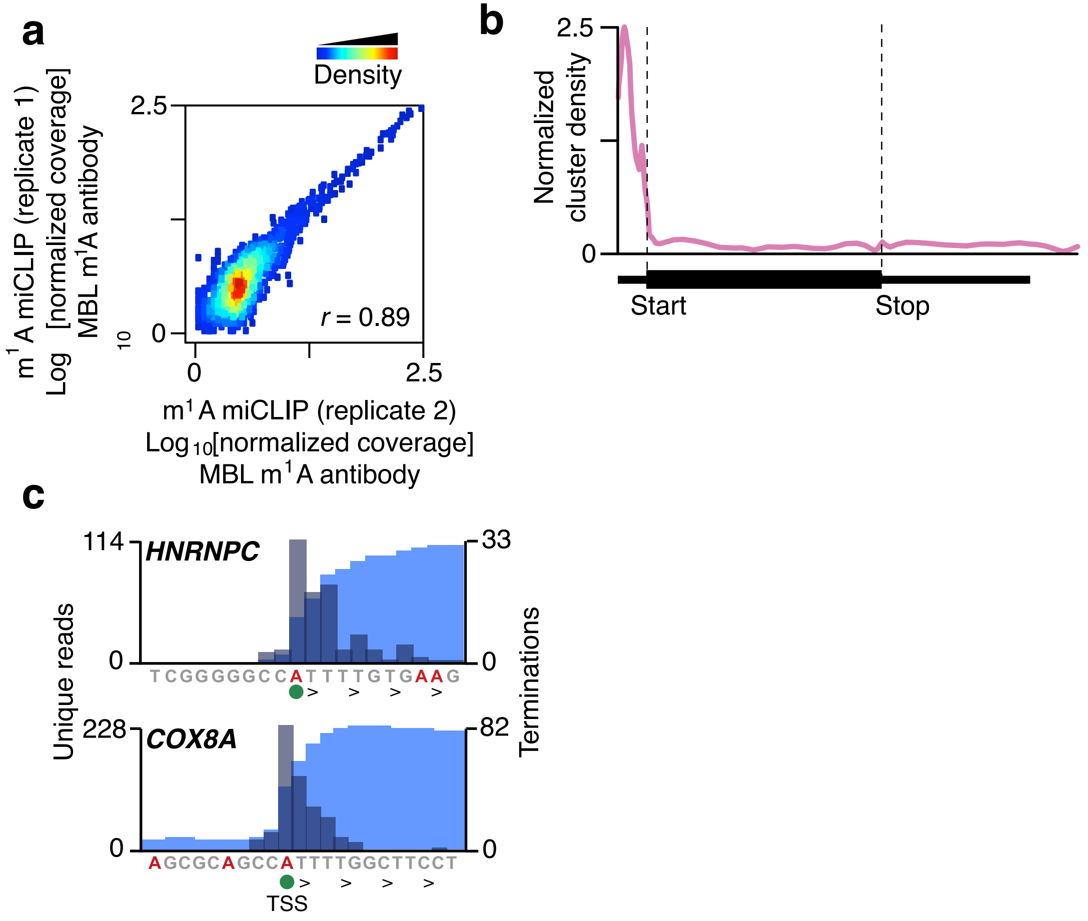

Supplementary Figure 5. m^{1}A-miCLIP with the MBL antibody enriches for reads close to transcription-start nucleotide

a, m^{1}A-miCLIP replicate correlation. The x and y axes of the scatter plots represent normalized read coverage in 100 nt bins on the human genome in replicate 1 (x) or replicate 2 (y) of m^{1}A-miCLIP performed in HEK293T cells. The replicates show highly reproducible read coverage across the genome (r = 0.89, Pearson correlation).

b, Metagene distribution of m^{1}A-miCLIP clusters in mouse brain transcripts. The density of m^{1}A-miCLIP clusters in mouse brain mRNAs was normalized to RNA-seq coverage (see **Methods**) and plotted on a virtual transcript (start, start codon; stop, stop codon). Like in human transcripts, m^{1}A-miCLIP clusters in mouse brain mRNA were highly enriched in the 5'UTR, and particularly, next to the transcription-start site.

c, Characterization of m^{1}A-miCLIP terminations at positions +2 and +5 relative to the putative site of antibody crosslinking. While many transcripts with m^{1}A-miCLIP clusters (light blue) in their 5'UTR showed enrichment of read terminations (dark blue) at the +1 position relative to the initiating nucleotide, certain transcripts had additional terminations between positions +2 and +5. We considered the possibility that these terminations reflect arrests of cDNA synthesis due to the presence of crosslinked antibody peptide on adenosine. Indeed, this was likely the case, as we observed that on certain transcripts like *HNRNPC* and *COX8A*, even though terminations occurred between positions +2 and +5 relative to the initiating adenosine, the only adenosine near terminations was at position 0 (green circles). Indeed, antibody crosslinks at transcription-start sites have been previously shown to generate read terminations at positions +2 to +5 relative to the transcription-start site, while the antibody crosslinking site was only at the transcription-start site[19].

Grozhik et al. Supplementary Figure 6

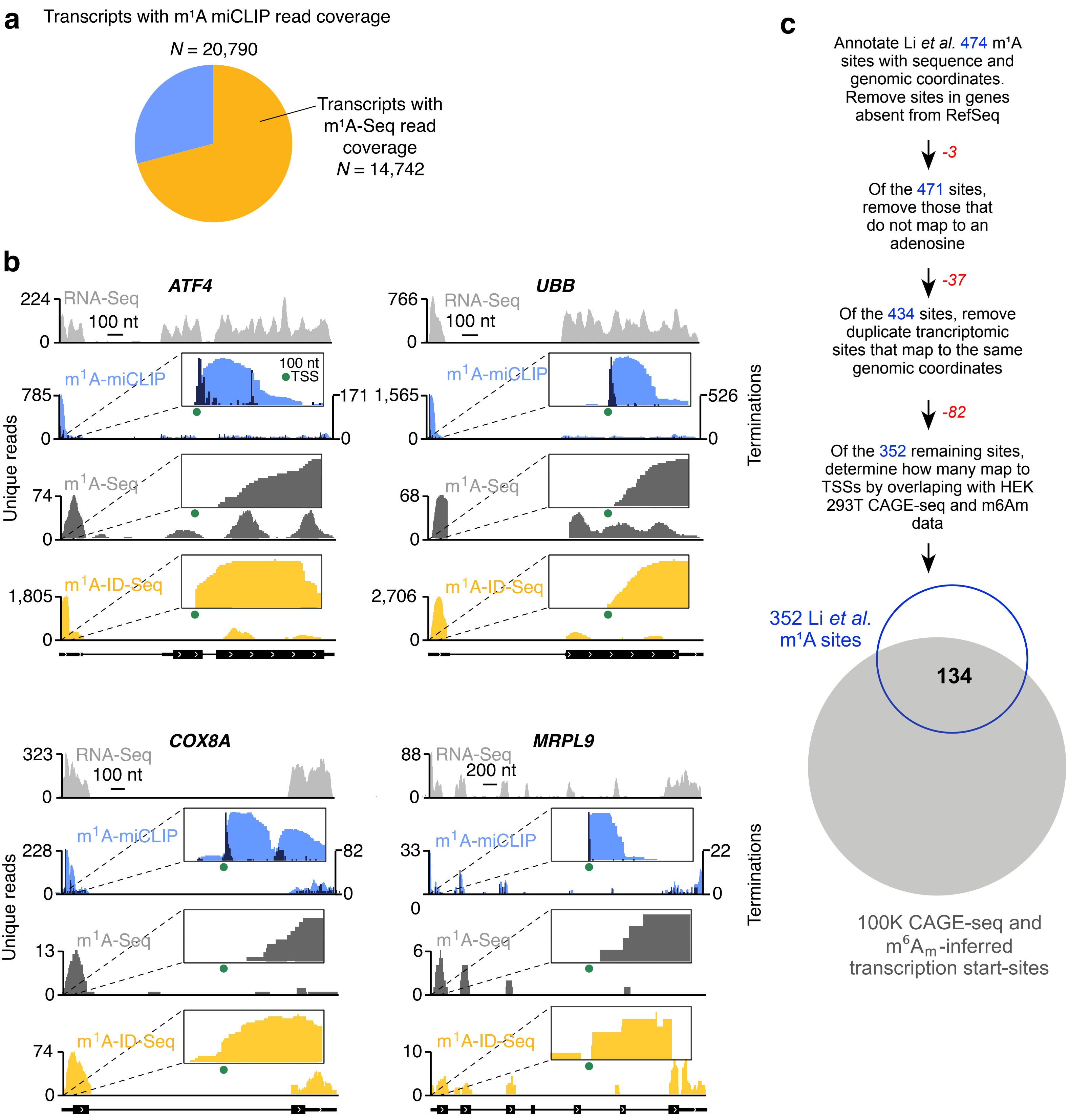

Supplementary Figure 6. Comparison of m^{1}A-miCLIP (MBL antibody) and earlier m^{1}A mapping strategies

a, m^{1}A-miCLIP and the earlier m^{1}A-Seq mapping strategy mark similar mRNAs. To compare m^{1}A-miCLIP with previous maps, we first identified unique transcripts containing m^{1}A-miCLIP or m^{1}A-Seq unique read coverage. Of the 20,790 transcripts containing m^{1}A-miCLIP coverage, more than ~70% also showed m^{1}A-Seq coverage. This demonstrates that overall, the m^{1}A-miCLIP profile of the transcriptome is similar to that of m^{1}A-Seq.

b, m^{1}A-miCLIP read distribution differs from earlier mapping methods. Here, we wanted to demonstrate that while m^{1}A-miCLIP (light blue) and earlier methods (m^{1}A-Seq, dark grey; m^{1}A-ID-Seq, yellow) have coverage on similar transcripts, m^{1}A-miCLIP marks transcription-start sites (green circles) due to differences in read distribution. The m^{1}A-miCLIP, m^{1}A-Seq, and m^{1}A-ID-Seq coverage of four HEK293T transcripts, *ATF4*, *UBB*, *COX8A*, and *MRPL9* (black bars; white arrows, direction of transcription), are shown. While all four transcripts demonstrated predominant peaks in their 5'UTRs in all three mapping protocols (see light grey RNA-Seq to note m^{1}A peak enrichment), m^{1}A-miCLIP clearly recognized adenine at transcription-start sites (see insets). This is because earlier mapping strategies did not preserve the full-length cDNA produced by reverse transcription of immunoprecipitated RNA fragments, resulting in a loss of detection of RNA 5' ends (see **Supplementary Figure 1a**). m^{1}A-miCLIP, on the other hand, preserves the full-length cDNA (see **Fig. 2a**), resulting in ready detection of transcription start sties. Taken together, these data demonstrate that the peaks identified by earlier m^{1}A mapping approaches are likely to be derived from adenine-containing extended cap structures in mRNAs.

c, Top, flow chart showing the filtering of non-validated m^{1}A sites from the Li *et al.* study. Bottom, Venn diagram showing overlap of final list of m^{1}A sites with transcriptional start sites inferred from CAGE-seq and m^{6}A$_m$ mapping data. Nearly 40% of putative m^{1}A sites are at transcriptional start sites.
