# Supplementary material for: Antibody cross-reactivity accounts for widespread appearance of m1A in 5’UTRs
Source: Nat Commun. 2019 Nov 12;10:5126. doi: 10.1038/s41467-019-13146-w (PMC6851129; doi:10.1038/s41467-019-13146-w)
Supplement: Supplementary file 2 — Reporting Summary [file 41467_2019_13146_MOESM2_ESM.pdf]

nature research

Corresponding author(s): Samie R. Jaffrey

Last updated by author(s): Jul 1, 2019

Reporting Summary

Nature Research wishes to improve the reproducibility of the work that we publish. This form provides structure for consistency and transparency in reporting. For further information on Nature Research policies, see Authors & Referees and the Editorial Policy Checklist.

Statistics

For all statistical analyses, confirm that the following items are present in the figure legend, table legend, main text, or Methods section.

n/a	Confirmed	
☐	☒	The exact sample size (*n*) for each experimental group/condition, given as a discrete number and unit of measurement
☒	☐	A statement on whether measurements were taken from distinct samples or whether the same sample was measured repeatedly
☐	☒	The statistical test(s) used AND whether they are one- or two-sided<br>*Only common tests should be described solely by name; describe more complex techniques in the Methods section.*
☒	☐	A description of all covariates tested
☒	☐	A description of any assumptions or corrections, such as tests of normality and adjustment for multiple comparisons
☒	☐	A full description of the statistical parameters including central tendency (e.g. means) or other basic estimates (e.g. regression coefficient) AND variation (e.g. standard deviation) or associated estimates of uncertainty (e.g. confidence intervals)
☐	☒	For null hypothesis testing, the test statistic (e.g. *F*, *t*, *r*) with confidence intervals, effect sizes, degrees of freedom and *P* value noted<br>*Give P values as exact values whenever suitable.*
☒	☐	For Bayesian analysis, information on the choice of priors and Markov chain Monte Carlo settings
☒	☐	For hierarchical and complex designs, identification of the appropriate level for tests and full reporting of outcomes
☒	☐	Estimates of effect sizes (e.g. Cohen's *d*, Pearson's *r*), indicating how they were calculated

Our web collection on statistics for biologists contains articles on many of the points above.

Software and code

Policy information about availability of computer code

Data collection	SRAtoolkit, ftp
Data analysis	STAR (version 2.5.3a) , Rsamtools Pileup (version 1.27.16), Flexbar (version 2.5) , pyCRAC suite (version 1.2.2) , Bowtie (version 1.1.2), MEME suite (version 4.11.4), MetaPlotR, Deeptools 3.0, R 3.4

For manuscripts utilizing custom algorithms or software that are central to the research but not yet described in published literature, software must be made available to editors/reviewers. We strongly encourage code deposition in a community repository (e.g. GitHub). See the Nature Research guidelines for submitting code & software for further information.

Data

Policy information about availability of data

All manuscripts must include a data availability statement. This statement should provide the following information, where applicable:

- Accession codes, unique identifiers, or web links for publicly available datasets
- A list of figures that have associated raw data
- A description of any restrictions on data availability

All data used in/generated from this paper are publicly available. Sequencing data have been deposited in GEO (GSE97909). The source data for figures 2e, 4b and 4c are provided in the Source Data File.

Field-specific reporting

Please select the one below that is the best fit for your research. If you are not sure, read the appropriate sections before making your selection.

☒ Life sciences ☐ Behavioural & social sciences ☐ Ecological, evolutionary & environmental sciences

For a reference copy of the document with all sections, see nature.com/documents/nr-reporting-summary-flat.pdf

Life sciences study design

All studies must disclose on these points even when the disclosure is negative.

Sample size	Sample size of two(human) or six (mouse) replicates per miCLIP (human) condition were based on previous miCLIP studies taking into account the goal of the experiment (exploratory) and practical considerations/limitations (large amounts of polyA RNA necessary). For dot blots sample size >= 3.
Data exclusions	No data exclusions
Replication	Experiments such as dot blots were repeated several times, on different days, by two different researchers with similar results. Our miCLIP results were corroborated by studies from other researchers.
Randomization	Not relevant to our study
Blinding	Investigator were aware of sample allocation during data acquisition and analysis

Reporting for specific materials, systems and methods

We require information from authors about some types of materials, experimental systems and methods used in many studies. Here, indicate whether each material, system or method listed is relevant to your study. If you are not sure if a list item applies to your research, read the appropriate section before selecting a response.

Materials & experimental systems

n/a	Involved in the study	
☐	☒	Antibodies
☐	☒	Eukaryotic cell lines
☒	☐	Palaeontology
☐	☒	Animals and other organisms
☒	☐	Human research participants
☒	☐	Clinical data

Methods

n/a	Involved in the study	
☒	☐	ChIP-seq
☒	☐	Flow cytometry
☒	☐	MRI-based neuroimaging

Antibodies

Antibodies used	The MBL m1A antibody in this study was a mouse monoclonal antibody (MBL D345-3). The Abcam m1A antibody was a rabbit monoclonal generated against m1A (ab208196).
Validation	Competitor assays with free nucleotide

Eukaryotic cell lines

Policy information about cell lines

Cell line source(s)	HEK293T (ATCC)
Authentication	Purchased from source without authentication
Mycoplasma contamination	Not tested
Commonly misidentified lines (See ICLAC register)	n/a

Animals and other organisms

Policy information about studies involving animals; ARRIVE guidelines recommended for reporting animal research

Laboratory animals	C57B6, male, 8 weeks mouse
Wild animals	Study did not involve wild animals
Field-collected samples	Study did not involve samples collected from the field.
Ethics oversight	Weill Cornell Medicine, IACUC approved the use of animals in this study.

Note that full information on the approval of the study protocol must also be provided in the manuscript.
