# Supplementary material for: Antibody cross-reactivity accounts for widespread appearance of m1A in 5’UTRs
Source: Nat Commun. 2019 Nov 12;10:5126. doi: 10.1038/s41467-019-13146-w (PMC6851129; doi:10.1038/s41467-019-13146-w)
Supplement: Supplementary file 3 — Description of Additional Supplementary Files [file 41467_2019_13146_MOESM3_ESM.docx]

**Description of Additional Supplementary Files**

File Name: Supplementary Data 1
Description: Sites identified by misincorporation mapping (filter: >500 coverage depth and at least 1% misincorporation rate)

File Name: Supplementary Data 2
Description: High-confidence sites identified by misincorporation mapping (filter: at least 5% misincorporation rate encompassing multiple transition types, to exclude potential heterozygous alleles and A-to-I editing sites)

File Name: Supplementary Data 3
Description: Misincorporation rates in our study of m1A sites identified in the Li and Safra studies

File Name: Supplementary Data 4
Description: m1A miCLIP clusters (score >/=20) in HEK 293T cell mRNAs

File Name: Supplementary Data 5
Description: m1A miCLIP clusters (score >/=20) in mouse brain mRNAs

File Name: Supplementary Data 6
Description: m1A miCLIP clusters in HEK293T cells that overlap RefSeq transcription start sites

File Name: Supplementary Data 7
Description: Primer and oligonucleotide sequences used in this study
